# Strong Replication Interference Between Hepatitis Delta Viruses in Human Liver Chimeric Mice

**DOI:** 10.3389/fmicb.2021.671466

**Published:** 2021-07-08

**Authors:** Katja Giersch, Lennart Hermanussen, Tassilo Volz, Annika Volmari, Lena Allweiss, Camille Sureau, John Casey, Jiabin Huang, Nicole Fischer, Marc Lütgehetmann, Maura Dandri

**Affiliations:** ^1^Department of Internal Medicine, University Medical Center Hamburg-Eppendorf, Hamburg, Germany; ^2^German Center for Infection Research (DZIF), Hamburg-Lübeck-Borstel-Riems Site, Hamburg, Germany; ^3^Institut National de la Transfusion Sanguine, Paris, France; ^4^Georgetown University Medical Center, Washington, DC, United States; ^5^Department of Medical Microbiology, Virology and Hygiene, University Medical Center Hamburg-Eppendorf, Hamburg, Germany

**Keywords:** HDV, genotypes, human liver chimeric mice, humanized mice, *in vivo*, replication, recombination, hepatitis delta

## Abstract

**Background:**

Hepatitis D Virus (HDV) is classified into eight genotypes with distinct clinical outcomes. Despite the maintenance of highly conserved functional motifs, it is unknown whether sequence divergence between genotypes, such as HDV-1 and HDV-3, or viral interference mechanisms may affect co-infection in the same host and cell, thus hindering the development of HDV inter-genotypic recombinants. We aimed to investigate virological differences of HDV-1 and HDV-3 and assessed their capacity to infect and replicate within the same liver and human hepatocyte *in vivo*.

**Methods:**

Human liver chimeric mice were infected with hepatitis B virus (HBV) and with one of the two HDV genotypes or with HDV-1 and HDV-3 simultaneously. In a second set of experiments, HBV-infected mice were first infected with HDV-1 and after 9 weeks with HDV-3, or vice versa. Also two distinct HDV-1 strains were used to infect mice simultaneously and sequentially. Virological parameters were determined by strain-specific qRT-PCR, RNA *in situ* hybridization and immunofluorescence staining.

**Results:**

HBV/HDV co-infection studies indicated faster spreading kinetics and higher intrahepatic levels of HDV-3 compared to HDV-1. In mice that simultaneously received both HDV strains, HDV-3 became the dominant genotype. Interestingly, antigenomic HDV-1 and HDV-3 RNA were detected within the same liver but hardly within the same cell. Surprisingly, sequential super-infection experiments revealed a clear dominance of the HDV strain that was inoculated first, indicating that HDV-infected cells may acquire resistance to super-infection.

**Conclusion:**

Infection with two largely divergent HDV genotypes could be established in the same liver, but rarely within the same hepatocyte. Sequential super-infection with distinct HDV genotypes and even with two HDV-1 isolates was strongly impaired, suggesting that virus interference mechanisms hamper productive replication in the same cell and hence recombination events even in a system lacking adaptive immune responses.

## Introduction

Around 20 million people worldwide are infected with the hepatitis delta virus (HDV) and recent reports suggested that the number of HDV-positive individuals may be even substantially higher (Chen et al., 2018). Its viral genome is a circular, negative-sense, single-stranded RNA, which forms a characteristic unbranched rod-like structure with paired nucleotides (Wang et al., 1986). HDV redirects the host RNA polymerase II to replicate via a double rolling-circle amplification process (Lai, 2005) leading to the intracellular accumulation of two additional RNAs: the antigenomic RNA (AG HDV RNA), which is an exact complement of the genomic RNA (G HDV RNA) and the smaller linear mRNA encoding for the only viral protein, the hepatitis delta antigen (HDAg). HDAg binds specifically to the HDV RNA and occurs in two different forms: the small HDAg (S-HDAg, 24 kDa) is important for virus replication, whereas the large variant (L-HDAg, 27 kDa) inhibits replication and promotes virus assembly (Chang et al., 1991; Wang et al., 1991). The L-HDAg is generated during virus replication by post-transcriptional RNA editing at adenosine 1012 (amber/W site), which is mediated by RNA-specific adenosine deaminase (ADAR). HDV requires the envelope proteins of the hepatitis B virus (HBV) in order to assemble into infectious particles and spread (Rizzetto et al., 1980; Freitas et al., 2014). Hence, HBV plays an essential role as helper virus for HDV transmission. Recently, the envelope proteins of other HBV-unrelated viruses (such as dengue virus, hepatitis C virus (HCV), or west nile virus) were shown to act as alternative helper viruses enabling coating of HDV *in vitro* (Perez-Vargas et al., 2019). However, clinical relevance for HCV/HDV co-infection appears unlikely, since three different studies analyzing chronic HCV-infected patients revealed only one case with detectable HDV RNA in the absence of HBV (Cappy et al., 2020; Chemin et al., 2020; Pfluger et al., 2021). Both HBV and HDV enter human hepatocytes via the sodium taurocholate co-transporting polypeptide (NTCP) (Yan et al., 2012) and active HDV infection can occur either upon simultaneous co-infection with HBV or as a super-infection in patients already infected with HBV. Chronic HDV infections are associated with severe liver disease and progression to cirrhosis, liver decompensation, hepatocellular carcinoma and death (Koh et al., 2019). HDV is classified into eight genotypes with distinct clinical courses (Le Gal et al., 2006; Botelho-Souza et al., 2017; Delfino et al., 2018). Sequence divergence among the genotypes is as high as 40% over the entire RNA genome with the greatest difference observed between HDV genotype 1 (HDV-1) and genotype 3 (HDV-3) (Deny, 2006). However, a recent systematic analysis of HDV sequences revealed highly conserved functional nucleotide and amino acid motifs across all genotypes, indicating strong conservatory constraints on the structure and function of the HDV genome and the HDAg proteins (Le Gal et al., 2017). HDV-1 is distributed around the world and causes a broad spectrum of chronic diseases. HDV-3 is almost exclusively detected in patients from the Amazonian region, where HDV infection is associated with particularly severe disease (Botelho-Souza et al., 2017). An *in vitro* study suggested that the severity of hepatic inflammation might be associated with the distinct efficacies of HDV genotypes to generate L-HDAg, package and secrete viral particles (Hsu et al., 2002). To date, most *in vitro* and *in vivo* experiments had been performed with a certain HDV-1 strain, which was obtained from an infected patient, serially passaged through chimpanzees and then in 1988 isolated and cloned after being transmitted to a woodchuck (Kuo et al., 1988, 1989). Ten years later, an HDV-3 isolate (Peru-1) was obtained from an 18-year-old man from Peru, who developed severe acute hepatitis, and cloned by Casey et al. (1993). However, little is known about the behavior and kinetics of different HDV isolates and divergent strains such as HDV-3 and HDV-1 *in vivo*. Such analyses may help understand differences in HDV pathogenesis and disease outcome.

Similar to other RNA viruses co-infections and inter-genotypic recombination between different HDV genotypes can occur. However, little is known about the ability of distinct HDV genotypes to infect and actively replicate within the same hepatocyte and, to our knowledge, recombination events have only been reported between HDV-1 and HDV genotype 2 (HDV-2), which are closely related (Wu et al., 1999; Wang and Chao, 2005; Sy et al., 2015). Co-infections including HDV-3, such as HDV1/HDV-3 co-infections in patients, have not been reported yet (Cicero et al., 2016), perhaps because HDV-3 is geographically rather isolated in the Amazonas region or because the high sequence divergence of HDV-3 to other genotypes prevents productive co-infection and inter-genotypic recombination. Virus interference limiting a productive co-infection of the same cell with HDV of distinct genotypes might play an important role in HDV epidemiology and geographical distribution.

The aim of this study was to investigate virological differences between two distinct cloned genotypes (HDV-1 and HDV-3) in human liver chimeric mice in the presence of HBV. Furthermore, to assess whether HDV-1 and HDV-3 can efficiently spread within the same liver and even replicate within the same hepatocyte, we performed both simultaneous and sequential super-infections experiments with HDV-1 and HDV-3 strains *in vivo* in a system lacking adaptive immune responses.

## Materials and Methods

### Generation of Humanized USG Mice

Human liver chimeric urokinase-type plasminogen activator (uPA)/severe combined immunodeficiency (SCID)/beige/interleukin-2 receptor gamma chain negative (IL2RG^–/–^) mice (short USG mice) were generated by transplanting one million thawed cryo-preserved human hepatocytes into homozygous USG mice as previously reported (Lutgehetmann et al., 2012). Repopulation rates were estimated by determining human serum albumin (HSA) in mouse sera (ELISA; Bethyl Laboratories, Biomol GmbH, Hamburg, Germany) and human beta-globin in mouse liver DNA (qRT-PCR; Taqman Gene Expression Assay Hs00758889_s1; Applied Biosystems, Carlsbad, CA, United States) (Lutgehetmann et al., 2012). Animals displaying high levels of human chimerism (>2 mg/ml HSA in serum) were used for the study. All mice were sacrificed at the end of the experiment (at different time-points as indicated in the results), blood was collected and liver specimens were snap-frozen in chilled isopentane and cryo-conserved at −80°C for further histological and molecular analyses. Mice were maintained under specific pathogen free conditions in accordance with institutional guidelines under approved protocols. All animal experiments were conducted in accordance with the European Communities Council Directive (86/609/EEC) and were approved by the City of Hamburg, Germany.

### Virus Generation and Infections

Human liver chimeric USG mice were either co-infected with HBV/HDV-1cc or HBV/HDV-3cc, or first infected with HBV and after 9 weeks super-infected simultaneously with HDV-1cc and HDV-3cc or HDV-1p. For each co-infection setting and for simultaneous HDV1/HDV3 super-infection, mice received a single peritoneal injection of cell culture derived HBV genotype D (1 × 10^7^ HBV genome equivalents/mouse, kindly provided by Dieter Glebe, Gießen, Germany), cell culture derived HDV-1 (HDV-1cc) (1 × 10^7^ HDV genome equivalents/mouse) and/or cell culture derived HDV-3 (HDV-3cc) (1 × 10^7^ HDV genome equivalents/mouse). For simultaneous super-infection with two different HDV-1 isolates chronic HBV-infected mice (>9 weeks) received patient derived HDV-1 (HDV-1p) (1 × 10^5^ HDV genome equivalents/mouse) and HDV-1cc (1 × 10^6^ HDV genome equivalents/mouse). For sequential HDV super-infection experiments, chronic HBV-infected mice (>9 weeks) were infected with HDV-3cc and after 9 weeks super-infected with HDV-1cc and vice versa - using the same inocula as described above. HBV-infected mice were also infected with a patient-derived HDV-1 strain (HDV-1p) and then super-infected with either HDV-3cc or HDV-1cc. Cell culture derived HDV-1cc and HDV-3cc particles were generated in HuH7 cells as previously described (Gudima et al., 2007). In brief, cells were transfected with 1 μg of the HDV recombinant plasmid pSVL(D3) for HDV-1 (kindly provided by John Taylor, Philadelphia, PA, United States) (Kuo et al., 1989) or pCMV3-Peru-1.2 for HDV-3 (Casey and Gerin, 1998) and 1 μg of the HBV envelope-expressing vector pT7HB2.7 using Fugene HD Transfection Reagent (Promega, Madison, United States). HDV-1p was isolated from a chronic HBV/HDV co-infected patient and passaged through humanized USG mice. 50 μl HBV/HDV-1p-positive mouse serum, which contained 7.3 × 10^6^ HBV DNA copies/ml and 1.3 × 10^8^ HDV RNA copies/ml, was used for infection. The inocula (1 × 10^7^ HDV genome equivalents/mouse) corresponded to a MOI of approximately 0.3 by estimating an average of 3 × 10^7^ human hepatocytes per mouse liver (Dandri et al., 2008).

### Virological Measurements in Serum and Liver

Viral DNA and RNA were extracted from serum samples using the QiAmp MinElute Virus Spin kit (Qiagen, Hilden, Germany) and from liver tissues using the MasterPure^TM^ Complete DNA and RNA Purification Kit (Epicentre, Madison, WI, United States) and the Qiagen RNeasy Mini Kit. HDV viremia and intrahepatic HDV RNA levels were determined by reverse transcription and qRT-PCR using the ABI Fast 1-Step Virus Master (Applied Biosystems, Waltham, MA, United States) and HDV Taqman primers and probes on an ABI Viia7 (Applied Biosystems, Waltham, MA, United States) as previously described (Giersch et al., 2019). Primers and probes recognized either all HDV genotypes (Ferns et al., 2011) or specifically HDV-1 (all isolates) (Mederacke et al., 2010), HDV-1cc (HDV1cc-fw: 5′-TCA CGG TAA AGA GCA TTG, HDV1cc-rv: 5′-TTC CCC TTC CAG AGA TTC-3′, HDV1cc probe: 5′-/56-FAM/CGT CCG CTT/ZEN/CCT GAG ACC), HDV-1p (HDV1p-fw: 5′-AGG AGT AAG ATC ATA GCG ATA-3′, HDV1p-rv: 5′-CTG CTC TCT TTG CTT TCC-3′, HDV1p probe: 5′-/56-FAM/CGC CTC GGT/ZEN/CTC CTC TAA-3′), or HDV-3 (HDV3-fw: 5′-GGT CCG TCG TTC CAT CCT TT-3′, HDV3-rv: 5′-GTA GCT CCC TCG GAT CGT TG-3′, HDV3 probe: 5′-/56-FAM/CTT ACC TCG TGG CCG GC/3BHQ_1/-3′). Primers and probes were designed to specifically detect the used HDV isolates and did not detect the respective other isolates, e.g., HDV-1 primers did not allow amplification of HDV-3 RNA, and HDV-3 primers did not detect HDV-1 RNA. HBV viremia and intrahepatic HBV pregenomic (pg) RNA levels were determined by qRT-PCR using specific primers and probe (Taqman Gene Expression Assay Pa03453406_s1, Applied Biosystems) (Malmstrom et al., 2012) under conditions previously described (Giersch et al., 2019). HBsAg quantification were performed on the Abbott Alinity I platforms (quantitative HBsAg kit, Abbott, Ireland, Diagnostic Division) after diluting the mouse serum 1:200 in the dilution serum (Abbott) as recommended by manufacturer. Alanine aminotransferase (ALT) was measured by using the Roche Cobas c111 System (Roche, Basel, Switzerland). For the measurements 5 μl of mouse serum was used. In HBV/HDV co-infection experiments, known amounts of an HDV containing plasmid were used as standard for serum HDV quantification. In all super-infection experiments, standard curves for HDV quantification were generated by extracting RNA from transfected HuH7 cell culture supernatant, which contained either HDV-1cc, HDV-, or HDV-3cc virions, using the QiAmp MinElute Virus Spin kit (Qiagen, Hilden, Germany). Residual plasmids were removed from the extracts by DNAse I (Epicentre/Lucigen, Madison, WI, United States) digestion followed by isopropanol precipitation. The HDV RNA standard concentration was then determined by qRT-PCR using HDV specific primers and probe (Ferns et al., 2011) and known amounts of an HDV containing plasmid. The HDV RNA standard curves for HDV-1 (all isolates), and HDV-3cc were prepared in 1:5 dilutions and can be found in Supplementary Figure 1. Known amounts of an HBV-containing plasmid was used as standard for serum HBV quantification in all experiments (Lutgehetmann et al., 2012). Steady-state levels of intracellular viral RNA amounts were normalized to human specific hGAPDH (Taqman Gene Expression Assay Hs99999905_m1, Applied Biosystems).

For genomic and antigenomic HDV RNA quantification, intracellular RNA was reverse transcribed using biotinylated genomic and antigenomic HDV RNA primers (Ferns et al., 2011) as previously described (Giersch et al., 2019). Biotinylated cDNA was purified with the MinElute PCR Purification Kit (Qiagen, Venlo, Netherlands) and isolated with dynabeads specifically interacting with biotin (Dynal Kilobase Binder Kit, Invitrogen, Carlsbad, CA, United States) following the manufacturer’s instructions. For qRT-PCR with purified biotinylated cDNA bound to dynabeads, HDV-specific primers and probes (Ferns et al., 2011) and the ABI Fast Advanced Master (Applied Biosystems) were used under the conditions described above.

### cccDNA Quantification

Total DNA was extracted from liver specimens using the Master Pure DNA purification kit (Epicentre/Lucigen) following the manufacturer’s instructions, which include a proteinase K digestion step. To remove rcDNA 1 μg of extracted liver DNA was digested with plasmid-safe ATP-dependent DNase (Epicentre/Lucigen) (30 U) at 37°C for 2 h. After heat inactivation (30 min at 70°C) and isopropanol precipitation, qRT-PCR with cccDNA-selective primers and probe (Malmstrom et al., 2012) (final concentration forward primer: 100 nmol/l, reverse primer: 800 nmol/l) was performed under the following conditions: 10 min initial denaturation at 95°C; 40 cycles: 1 s at 95°C, 1 min at 65°C). cccDNA copies were normalized to the number of human hepatocytes, which was estimated by measuring the single copy gene human hemoglobin beta (Taqman Gene Expression Assay Hs00758889_s1, Applied Biosystems). Known amounts of cccDNA containing plasmid and human genomic DNA (Roche Applied Science, Mannheim, Germany) were used as a standard for quantification.

### Sequencing

For HDV genome sequencing of the amber/W site (position 1012) cDNA was synthesized with the Transcriptor First Strand cDNA Synthesis Kit (Roche, Mannheim, Germany) using random hexamer primer according to the manufacturer’s instructions. HDV region R1 [location: 305–1285, product size: 980 bp, according to the numeration of Wang et al. (1986)) were amplified by PCR using cDNA, respective primers (R1 fw 305-327: CCA GAG GAC CCC TTC AGC GAA C, R1 rv 1285-1261: GAA GGA AGG CCC TCG AGA ACA AGA) (Ivaniushina et al., 2001] and a Red-Taq Polymerase (Sigma-Aldrich, St. Louis, United States) under the following conditions: 4 min initial denaturation at 94°C; 45 cycles: 1 min at 94°C, 1 min at 62°C, and 2 min at 72°C. PCR product length was analyzed on a 0.8% agarose gel and DNA fragments were extracted with the MinElute PCR Purification Kit (Qiagen) as recommended by the manufacturer. The forward and reverse strand was sequenced with Sanger sequencing (Mix2seq kit) performed by Eurofins Genomics (Ebersberg, Germany).

### RNA Library Preparation/Next Generation Sequencing (NGS)

RNA Illumina NGS libraries were prepared from each sample using SMARTer Stranded Total RNA-Seq Kit v2 - Pico Input Mammalian (Takara Bio Europe, Saint-Germain-en-Laye, France). All libraries were multiplex-sequenced on an Illumina MiSeq instrument (300 cycles, PE protocol). Total numbers of 3,249,720 and 3,193,042 paired-end 151-nucleotide (nt) reads for the samples HDV1/3-liver, and HDV1/1-liver were generated. Adapter sequences of the reads and bases with a score of less than Q30 were trimmed, and any reads shorter than 40 nt removed using Trimmomatic v0.36 (Bolger et al., 2014). The high-quality paired-end reads were mapped to the host genome (10 mm, GCA_000001635.2) using the Bowtie2 package (Langmead and Salzberg, 2012). The unaligned reads were converted to fastq files using the SamTofastq tool in Picard tools. The tool FLASH (Magoc and Salzberg, 2011) was used to stitch the paired-end reads since over 70 percent of the reads overlap the reads generated from the opposite end of the same DNA fragment. The resulting stitched sequences together with the remaining paired-end reads were used as input data for the SPAdes assembler (v3.13.0) (Bankevich et al., 2012).

### Expression of Human Specific Interferon Stimulated Genes (ISGs)

To determine intrahepatic expression levels of human specific interferon stimulated genes (ISGs) in USG mice, liver RNA was extracted as described above and cDNA was synthesized with the Transcriptor First Strand cDNA Synthesis Kit (Roche, Mannheim, Germany) using oligo-dT primer according to the manufacturer’s instructions. qRT-PCR was performed with the ABI Fast Advanced Master (Applied Biosystems) in an ABI Viia7 (Applied Biosystems). The following Taqman Gene Expression Assays from Applied Biosystems containing human specific primers and probe, which do not cross-react with murine signals, were used: hMxA (Hs00895608_m1), hISG15 (Hs00192713_m1), hCXCL10 (Hs00171042_m1), hOAS1 (Hs00973637_m1), hHLA-E (Hs03045171_m1, and hCASP8 (Hs01018151_m1). The human housekeeping genes hGAPDH (Hs99999905_m1) and hRPL30 (Hs00265497_m1) were used for normalization.

### Immunofluorescence Stainings

Cryostat sections of chimeric mouse livers were stained as previously described (Lutgehetmann et al., 2012). Briefly, sections were fixed with acetone and incubated with mouse anti-CK18 (1:400, Dako, Glostrup, Denmark), rabbit anti-HBcAg (1:2000, Dako), mouse HLA-ABC (1:50, Antibodies-online, Aachen, Germany), and human anti-Delta (anti-HDAg-positive human serum, 1:8,000). Specific signals were visualized with Alexa 488-, 555-, or 633-labeled secondary antibodies (Invitrogen, Darmstadt, Germany). To enhance the HBcAg staining an anti-rabbit horseradish-peroxidase conjugated secondary antibody (Jackson Immunoresearch, Suffolk, United Kingdom) and the TSA Fluorescein System (Perkin Elmer, Jügesheim, Germany) were used. Nuclear staining was achieved by Hoechst 33258 (1:20,000 diluted, Invitrogen, Waltham, MA, United States). Stained sections were then mounted with fluorescent mounting media (Dako) and analyzed with the fluorescence microscope BZ8710 (Keyence, Osaka, Japan) using the same settings for the different experimental groups. The percentages of HDAg-positive human hepatocytes were estimated as previously described (Lutgehetmann et al., 2012) and by using 2–5 visual fields (displaying an average of 500 human hepatocytes) per mouse liver.

### RNA *in situ* Hybridization (RNAScope)

RNA *in situ* hybridization was performed on paraformaldehyde-fixed, cryo-preserved liver sections using the RNAScope Fluorescent Multiplex Kit (Advanced Cell Diagnostics, ACD, Hayward, CA, United States) according to the manufacturer’s instructions and as previously described (Allweiss et al., 2016). Briefly, liver sections were fixed with 4% paraformaldehyde, dehydrated with ethanol and pretreated with Pretreat 4 (Pretreatment Kit, ACD) for 30 min. Liver sections were then incubated with RNAScope target probes, which specifically bind HBV pgRNA (assay number: 442741), HDV-1 (475311) or HDV-3 (478121-C2) AG HDV RNA, hISG15 (450921-C3), or hMxA (403831-C3) for 2 h at 40°C (HybEZ oven, ACD). DAPI staining was performed to visualize nuclei. Stained sections were analyzed by fluorescence microscopy (Biorevo BZ-9000, Keyence) using a 60 × /1.40 NA oil objective. Merged z stack images were prepared using the same settings for all groups. The percentages of HDV RNA positive human hepatocytes were estimated by using 3 visual fields (displaying an average of 500 human hepatocytes) per mouse liver.

### Western Blot

Protein lysates were obtained by extracting liver tissue with T-Per Tissue Protein Extraction Reagent (Pierce, Rockford, United States) supplemented with NaF (10 mM), EDTA (2 mM), benzamidine (10 mM), PMSF (1 mM), leupeptin (1 μg/ml), Na3VO4 (2 mM), and aprotinin (1,5 μg/ml). Protein content was measured by Pierce BCA Protein Assay Kit (Thermo Scientific, Rockford, United States) following the manufacturer’s instructions. For immunoblotting, 20 μg of protein extracts were denaturated at 95°C, separated on a 12% sodium dodecyl sulfate-polyacrylamide gel (NuView Precast gels, Peqlab, Erlangen, Germany) and blotted onto a nitrocellulose membrane (Hybond ECL Nitrocellulose Membrane, GE Healthcare, Buckinghamshire, United Kingdom). Small and large hepatitis delta antigens (S-HDAg, L-HDAg) were detected using a highly diluted human anti-Delta antibody (anti-HDAg-positive human serum (1:2,000). An anti-human horseradish-peroxidase conjugated secondary antibody (Jackson Immunoresearch, Suffolk, United Kingdom) was used in a dilution of 1:10,000. Signals were visualized with Super Signal West Dura Chemiluminescent Substrate (Pierce) and the Molecular Imager ChemiDoc XRS System (Bio-Rad Laboratories, Hercules, United States).

### Statistics

Statistical analyses were performed with the GraphPad Prism 9 software. For group-wise comparisons, the non-parametric Mann-Whitney *U* test was applied. For correlations the Spearman test was used. *P* values < 0.05 were considered statistically significant.

## Results

### HDV-3cc Infected Mice Show Higher Intrahepatic Amounts of HDV RNAs and HDAg Than Animals Infected With a HDV-1cc Strain

**FIGURE 1:**
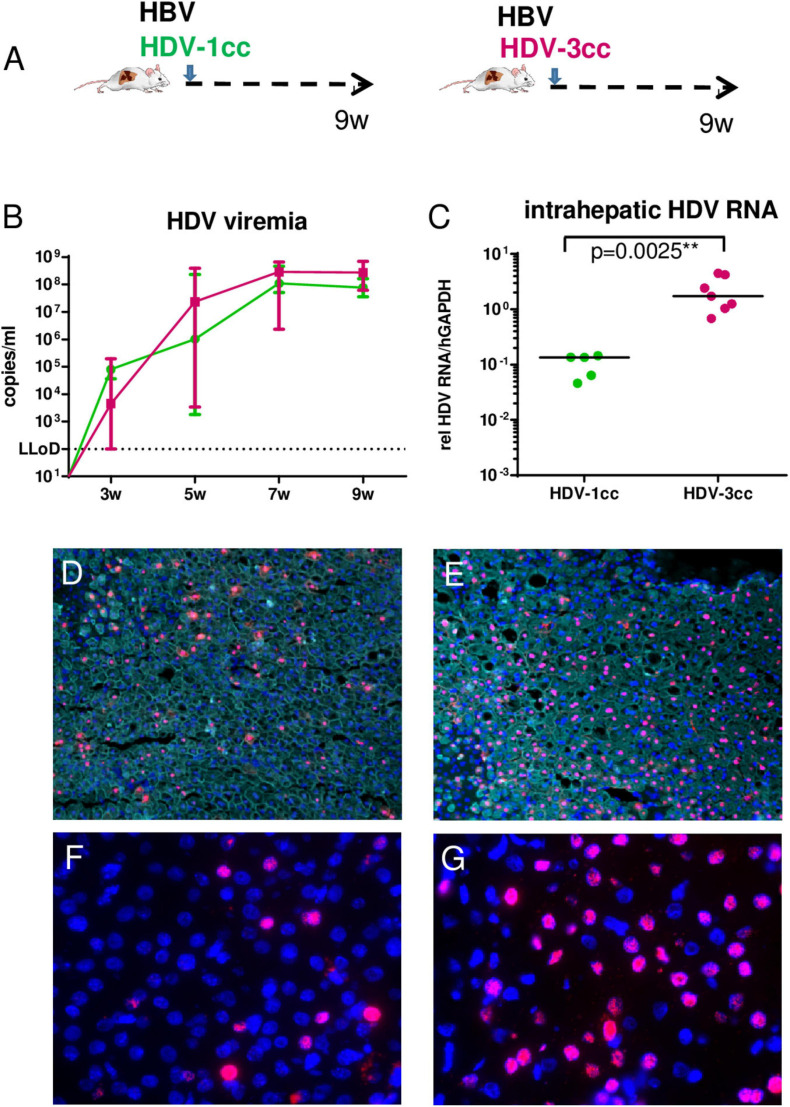
HDV in mice co-infected with HBV and HDV-1cc or HDV-3cc. **(A)** Experimental setting. qRT-PCR measurements (HDV primers/probe recognize all genotypes) of serum HDV RNA (quantification with plasmid standard) **(B)** and liver HDV RNA (normalized to housekeeping gene hGAPDH) **(C)** in HBV/HDV-1cc (green) and HBV/HDV-3cc (red) co-infected mice 9 weeks post infection. Results are expressed as median ± range **(B)**, the bar shows median levels **(C)**. **p < 0.01. Immunofluorescence staining of HDAg (red) and CK18 (detecting human hepatocytes, aqua) in HBV/HDV-1cc **(D)** and HBV/HDV-3cc **(E)** co-infected mice at the end of the experiment. Nuclei are stained with Hoechst 33258 (blue). RNA *in situ* hybridization staining of AG HDV RNA (red) in HBV/HDV-1cc **(F)** and HBV/HDV-3cc **(G)** co-infected mice at the end of the experiment. Nuclei are stained with dapi (blue).

**FIGURE 2:**
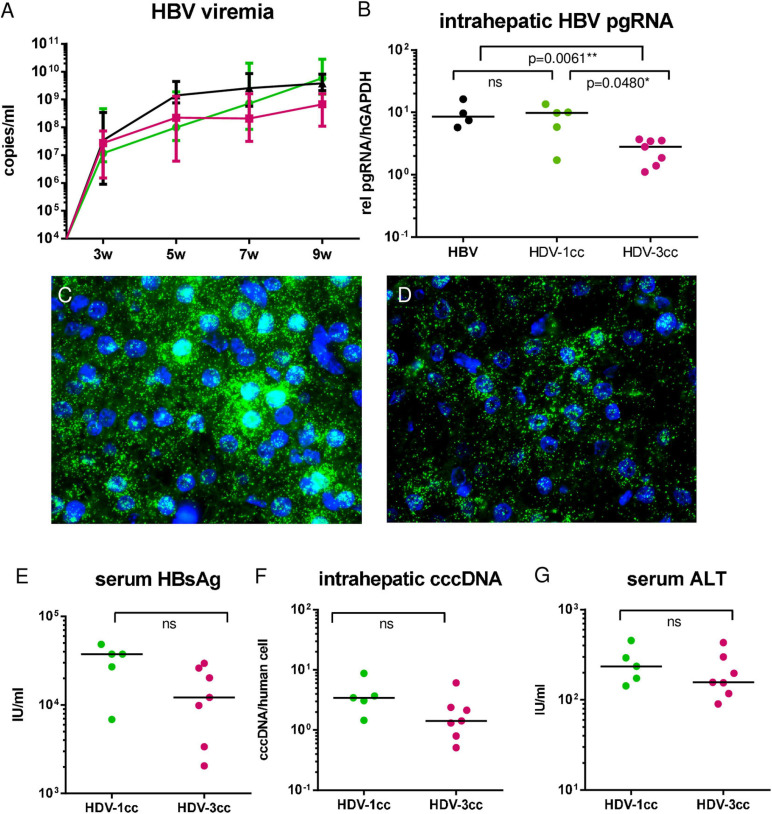
HBV in mice co-infected with HBV and HDV-1cc or HDV-3cc. qRT-PCR measurements of serum HBV DNA (quantification with plasmid standard) **(A)** and liver pregenomic HBV RNA (normalized to housekeeping gene hGAPDH) **(B)** in HBV/HDV-1cc (green line or dots) and HBV/HDV-3cc (red line or dots) co-infected mice 9 weeks post infection compared to stable HBV mono-infected mice (black line or dots). Results are expressed as median ± range **(A)**, the bar shows median levels **(B)**. *p < 0.05, **p < 0.01. RNA *in situ* hybridization (RNAScope) staining of pregenomic HBV RNA (green) in HBV/HDV-1cc **(C)** and HBV/HDV-3cc **(D)** co-infected mice at the end of the experiment. Nuclei are stained with dapi (blue). Serum HBsAg levels **(E)**, intrahepatic cccDNA levels (normalized to human beta globin) **(F)** and serum ALT levels **(G)** in HBV/HDV-1cc and HBV/HDV-3cc co-infected mice at the end of the experiment. The bar shows median levels **(E–G)**.

To comparatively assess infection and replication capacities of both cell culture (cc)-derived HDV strains, humanized USG mice were infected with HBV and either HDV-3cc (*n* = 7) or HDV-1cc (*n* = 5) and viral parameters were compared (Figure 1A). HDV viremia increased during the first 7 weeks of infection and reached stable levels of median 3 × 10^8^ HDV RNA copies/ml in HBV/HDV-3cc co-infection and of median 8 × 10^7^ HDV RNA copies/ml in HBV/HDV-1cc co-infected mice (Figure 1B). Nine weeks post infection (p.i.) median HDV levels were slightly higher in serum (0.4log, *p* = 0.0303) (Figure 1B) and clearly increased in the liver (1.1-log, *p* = 0.0025) of HBV/HDV-3cc-infected mice, compared to HBV/HDV-1cc-infected animals (Figure 1C). Consistent with the increased intrahepatic HDV RNA levels, the amount of HDAg positive human hepatocytes appeared higher in HBV/HDV-3cc co-infected mice (approximately 70 vs. 20% in HDV-1cc co-infection) (immunofluorescence staining, Figures 1D,E). Moreover, a clearly higher number of human cells was positive for genomic (G) and antigenomic (AG) HDV RNA in HDV-3cc infection compared to HDV-1cc infection (RNA *in situ* hybridization, Figures 1F,G). The increased levels of G and AG HDV RNA in HDV-3cc-infected mouse livers were also confirmed by performing a specific qRT-PCR assay using biotinylated reverse transcription primers and Dynabeads (see section “Materials and Methods”; Supplementary Figure 2A). RNA Sanger sequencing of the amber/W site revealed that the HDV RNA encoding for the S-HDAg (ATC) tends to be dominant in serum of HDV-3cc-infected mice, while human hepatocytes infected with HDV-1cc preferentially generated HDV RNA encoding for the L-HDAg (ACC) (Supplementary Figure 2B). However, the protein expression of intrahepatic S- and L-HDAg determined by western blot was very similar in HBV/HDV-1cc- and HBV/HDV-3cc-infected animals (Supplementary Figure 2C). The slightly different migration properties of HDV-1 and HDV-3 HDAgs likely occur due to different amino acid sequences and were also observed in an *in vitro* study comparing these two genotypes (Casey and Gerin, 1998). In line with previous findings (Lutgehetmann et al., 2012), the increased efficacy of HDV-3cc to infect humanized livers was associated with certain levels of HBV suppression, as indicated by the lower HBV viremia (1.0-log, *p* = 0.0051, Figure 2A) and intrahepatic HBV pgRNA levels (0.6-log, *p* = 0.0480, Figure 2B) determined in mice co-infected with HDV-3, compared to HBV-mono-infected and HBV/HDV-1cc co-infected mice. The development of HBV viremia was also slightly delayed in HBV/HDV-1 co-infected mice. However, intrahepatic HDV RNA levels remained lower in these mice and liver HBV pgRNA levels appeared comparable in HBV mono-infected and HBV/HDV-1 infected mice at 9 weeks p.i. (Figures 2A,B). Likewise, RNA *in situ* hybridization staining demonstrated substantially lower amounts of HBV pgRNA in livers of HBV/HDV-3cc-infected than in HBV/HDV-1cc-infected animals (Figures 2C,D). Circulating HBsAg levels (Figure 2E) and intrahepatic amounts of covalently closed circular (ccc) DNA (Figure 2F) did not differ significantly between HDV-1 and HDV-3 infected animals, although levels appeared lower in mice receiving HDV-3, further indicating HDV-mediated suppression of HBV. Furthermore, we investigated whether HDV-1cc and HDV-3cc infection leads to different cytotoxic effects on human hepatocytes of USG mice. In livers of both HDV-1cc and HDV-3cc-infected mice the apoptosis marker human caspase 8 was not significantly induced compared to uninfected controls (Supplementary Figure 3A, qRT-PCR). Immunofluorescence staining demonstrated very low and similar numbers of caspase 3 positive human hepatocytes in both co-infection settings (data not shown). Levels of serum ALT, which increase during liver injury, were comparable in HBV/HDV-3cc and HBV/HDV-1cc-infected mice (Figure 2G). In line, human serum albumin (HSA) levels, which reflect the amount of human hepatocytes in chimeric mouse livers, remained stable throughout the course of infection with both HDV-1cc and HDV-3cc (Supplementary Figure 3B), indicating that neither HBV/HDV-1cc nor HBV/HDV-3cc co-infections promote detectable cell death in USG mice.

**FIGURE 3:**
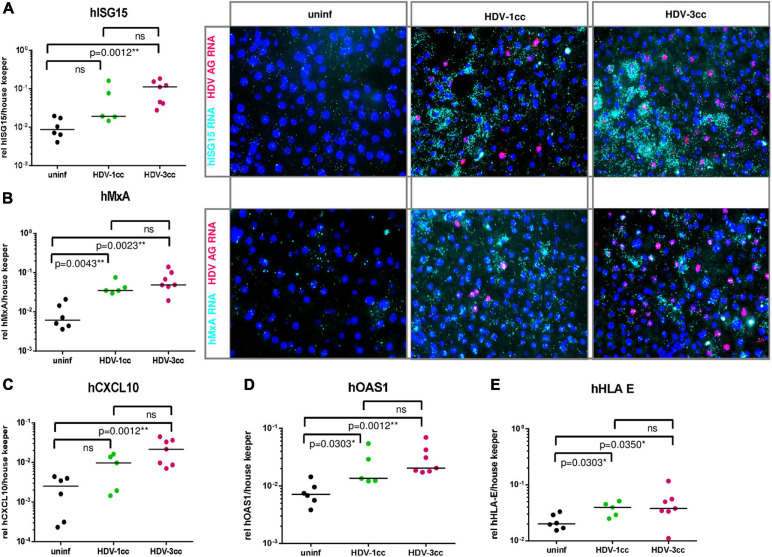
Human specific ISGs and chemokines in mice co-infected with HBV and HDV-1cc or HDV-3cc. **(A–E)** qRT-PCR measurements (normalized to median of housekeeping genes hGAPDH and hRPL30) **(A left, B left, and C–E)** and RNA *in situ* hybridization staining **(A right, B right)** of human ISG15 **(A)**, MxA **(B)**, CXCL10 **(C)**, OAS1 **(D)**, and HLA-E **(E)** in livers of HBV/HDV-1cc and HBV/HDV-3cc co-infected mice 9 weeks post infection compared to uninfected mice. The bar shows median levels. *p < 0.05, **p < 0.01. RNA *in situ* hybridization stainings show hISG15 RNA and hMxA RNA in aqua and HDV AG RNA in red. Nuclei are stained with dapi (blue).

Interestingly, mRNA expression levels of human specific interferon simulated genes (hISGs) such as hMxA, hOAS1 and hHLA-E did not differ significantly between HBV/HDV-3cc and HBV/HDV-1cc co-infected mice, but clearly increased compared to uninfected animals on a global level (qRT-PCR) and on a single cell level (RNA *in situ* hybridization) (Figure 3). The expression of hISG15 and hCXCL10 tended to be more enhanced in HDV-3cc-infected mice compared to HDV-1cc-infected animals (Figure 3) and such ISG increase correlated with intrahepatic HDV RNA levels (Supplementary Figure 4). Of note, an induction of hISGs was not exclusively observed in HDV AG RNA-positive or neighboring human cells, but also in further distant HDV-negative bystander human hepatocytes (Figure 3). Triple staining of HLA-ABC, HBcAg and HDAg indicated that the HDV-negative cells that show an induction of ISGs were either HBcAg-positive or negative for both of these HDV and HBV infection markers (Supplementary Figure 5). The underlying mechanisms mediating this rather “global sensing” of the infection needs to be investigated in future studies. Overall, this HDV-3cc strain demonstrated a higher infection efficiency in USG mice, as displayed by increased HDV viremia, intrahepatic HDV RNA levels and increased HBV suppression capacities compared to HDV-1cc, while levels of apoptosis and liver injury markers were comparable between HDV genotypes in human liver chimeric mice that lack functional NK, B and T cells.

### HDV-3cc/HDV-1cc Co-infection Occurs in USG Mice but Not at the Single Cell Level

**FIGURE 4:**
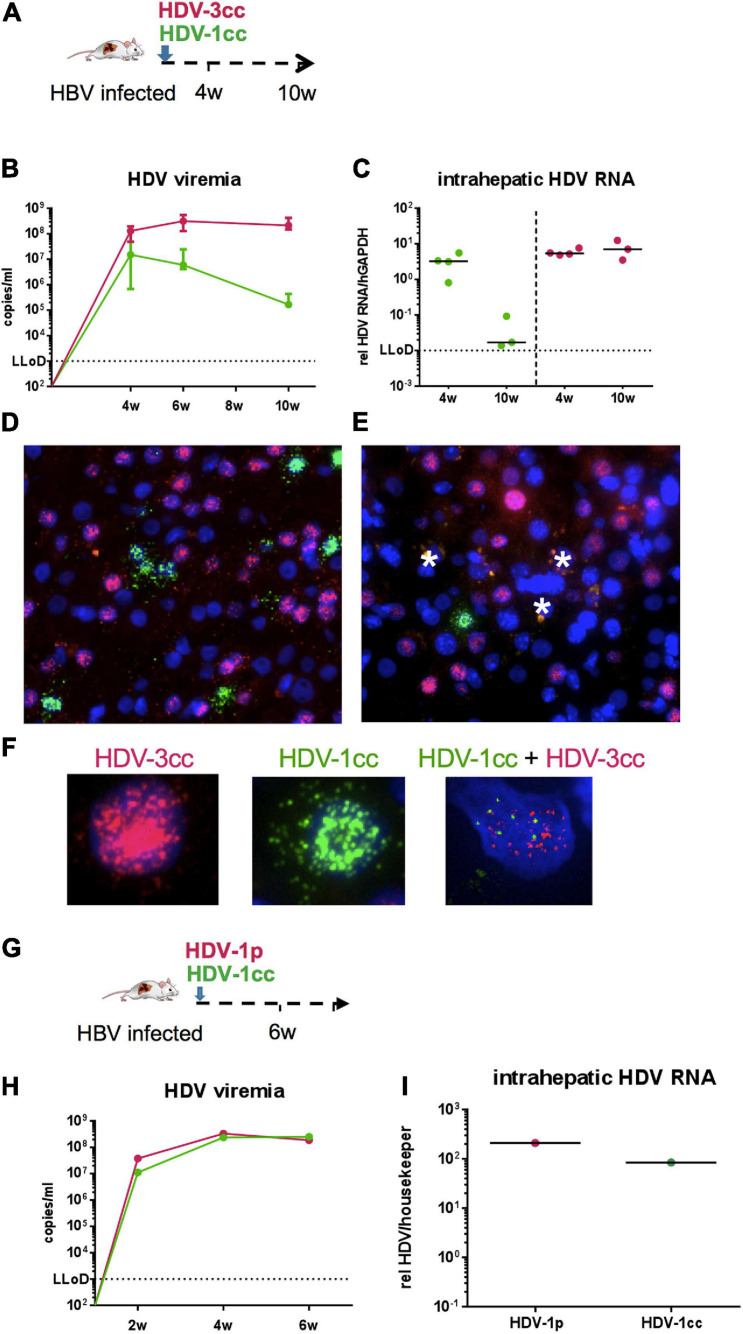
HDV in chronic HBV-infected mice simultaneously super-infected with HDV-1cc and HDV-3cc or with HDV-1cc and HDV-1p. **(A)** Experimental setting of HDV-1/HDV-3 double infection. **(B,C)** qRT-PCR measurements (genotype specific HDV primers/probe for HDV-1 and HDV-3 were used) of serum HDV RNA (quantification with cell culture derived RNA standard) at different time-points **(B)** and liver HDV RNA (normalized to housekeeping gene hGAPDH) 4 and 10 weeks post super-infection with HDV **(C)**. Results are expressed as median ± range **(B)**, the bar shows median levels **(C)**. HDV-1cc RNA levels are depicted in green (line or dots) and HDV-3cc RNA levels in red (line or dots). **(D,E)** RNA *in situ* hybridization (RNAScope) staining of HDV-1cc (green) and HDV-3cc AG HDV RNA (red) in HDV-1cc/HDV-3cc-infected mice 4 weeks **(D)** and 10 weeks **(E)** post super-infection. Phagocytic remnants have an autofluorescence and are marked with a white star. **(F)** Examples of HDV-3cc AG (red, left), HDV-1cc AG (green, middle), and HDV1/3 AG double positive human hepatocytes in mice infected with both HDV genotypes. Nuclei are stained with dapi (blue). **(G)** Experimental setting of HDV-1cc/HDV-1p double infection. **(H,I)** qRT-PCR measurements (strain specific HDV primers/probe for HDV-1cc and HDV-1p were used) of serum HDV RNA (quantification with cell culture derived RNA standard) at different time-points **(H)** and liver HDV RNA (normalized to housekeeping gene hGAPDH) 6 weeks post super-infection with HDV **(I)**. HDV-1cc RNA levels are depicted in green (line or dots) and HDV-1p RNA levels in red (line or dots).

To explore the ability of these two HDV genotypes to co-infect human hepatocytes *in vivo*, stably HBV-infected humanized mice (*n* = 7, median HBV viremia: 5 × 10^9^ copies/ml) were simultaneously infected with HDV-3cc and HDV-1cc (Figure 4A). Four weeks p.i., both HDV genotypes were detected in serum and livers of USG mice by using HDV-1 and HDV-3 specific qRT-PCRs, which did not detect the respective other HDV genotype (Figures 4B,C). Interestingly, HDV-3cc viremia reached maximum levels already at 4 weeks p.i. and remained stable until the end of the experiment at 10 weeks p.i. (Figure 4B). In contrast, HDV-1cc RNA in serum was slightly lower (0.9-log) compared to HDV-3cc levels at 4 weeks p.i., but strongly decreased (more than 3-log) in the following weeks (Figure 4B). In line with the viremia levels, intrahepatic HDV-3cc RNA was already at high levels at 4 weeks p.i. and at similar levels in mice sacrificed at 10 weeks p.i., while the HDV-1cc RNA levels within these HBV/HDV-1cc/HDV-3cc co-infected mouse livers at 10 weeks p.i. appeared clearly lower than in livers of mice sacrificed at 4 weeks p.i. (Figure 4C). Strain-specific RNA *in situ* hybridization indicated a similar number of HDV-1cc and HDV-3cc AG RNA-positive human hepatocytes at week 4 p.i. in livers of HBV/HDV-1cc/HDV-3cc-infected mice (Figure 4D). At 10 weeks p.i. clearly lower amounts of HDV-1cc AG RNA-positive human hepatocytes (1%) were detected compared to 4 weeks p.i. (Figure 4E), indicating that HDV-3cc became the predominant strain in HBV/HDV-1cc/HDV-3cc-infected mice (66.7% HDV-3cc AG RNA-positive). Strikingly, AG HDV RNAs of HDV-1cc and HDV-3cc could very rarely (less than 0.1%) be detected in the same hepatocyte at any of the time-points observed (Figure 4F), suggesting that these HDV genotypes do not replicate efficiently within the same cell. HBV viremia remained stable for the first 4 weeks after simultaneous super-infection with HDV-1cc and HDV-3cc but decreased in the following 6 weeks (0.8-log, *p* = 0.0167), while HBV mono-infected mice maintained stable HBV DNA viremia levels until the end of the experiment (Supplementary Figure 6A). Also intrahepatic HBV pgRNA levels were still high - and comparable to stable HBV mono-infected mice - at 4 weeks post HDV super-infection but appeared lower at 10 weeks post HDV super-infection (1.2-log decrease, Supplementary Figure 6B).

To comparatively assess the capacity of two different HDV strains with less divergent genome sequences to infect the livers of humanized mice, we super-infected one stable HBV-infected mice with HDV-1cc and another HDV-1 isolate derived from a chronic HBV/HDV infected patient (HDV-1p) (Figure 4G). 6 weeks after simultaneous HDV-1cc/HDV-1p super-infection, high and comparable levels of HDV RNA were measured in serum and liver by employing a strain specific qRT-PCR (which did not cross-react with the respective other HDV-1 isolate) (Figures 3H,I). Due to the high sequence similarity between HDV-1cc and HDV-1p, the RNA *in situ* hybridization assay could not be performed, thus hindering analyses of co-infection events at single-cell level. However, NGS analysis revealed that within the observation time, no recombination events were detected between HDV-1cc and HDV-3cc, or HDV-1cc and HDV1p *in vivo* (data not shown). These data suggest that concomitant infection of the same hepatocyte with two actively replicating HDV strains may not be a common event *in vivo*.

### Sequential Super-Infection With HDV-3cc and HDV-1cc (and Vice Versa) Was Strongly Impaired *in vivo*

**FIGURE 5:**
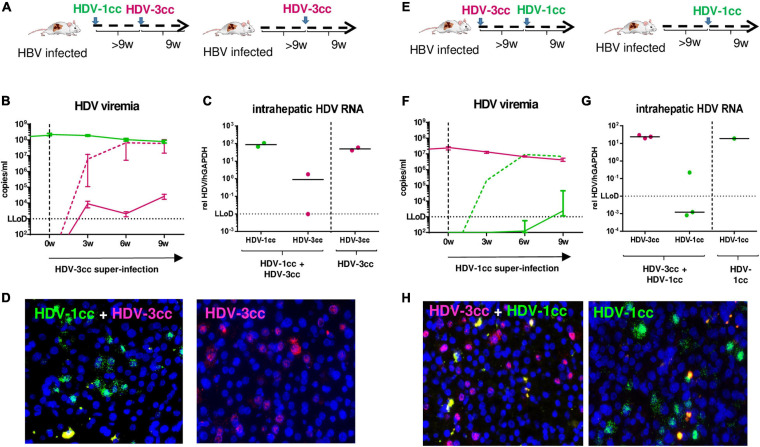
HDV in chronic HBV-infected mice sequentially super-infected with HDV-1cc and HDV-3cc. **(A)** Experimental setting (first HDV-1cc, then HDV-3cc; control mice were super-infected with HDV-3cc only). **(B,C)** qRT-PCR measurements (genotype specific HDV primers/probe for HDV-1 and HDV-3 were used) of serum HDV RNA (quantification with cell culture derived RNA standard) at different time-points **(B)** and liver HDV RNA (normalized to housekeeping gene hGAPDH) at the end of experiment **(C)**. HDV-1cc RNA levels are depicted in green (line or dots) and HDV-3cc RNA levels in red (line or dots). The dashed red line shows HDV viremia in control mice (HDV-3cc only). Results are expressed as median ± range **(B)**, the bar shows median levels **(C)**. **(D)** RNA *in situ* hybridization (RNAScope) staining of HDV-1cc (green) and HDV-3cc AG HDV RNA (red) in HDV-1cc/HDV-3cc sequential super-infected (**left**) and HDV-3cc control mice (**right**) at the end of the experiment. Nuclei are stained with dapi (blue). **(E)** Experimental setting (first HDV-3cc, then HDV-1cc; control mice were super-infected with HDV-1cc only). **(F,G)** qRT-PCR measurements (genotype specific HDV primers/probe for HDV-1 and HDV-3 were used) of serum HDV RNA (quantification with cell culture derived RNA standard) at different time-points **(F)** and liver HDV RNA (normalized to housekeeping gene hGAPDH) at the end of experiment **(G)**. HDV-1cc RNA levels are depicted in green (line or dots) and HDV-3cc RNA levels in red (line or dots). The dashed green line shows HDV viremia in control mice (HDV-1cc only). Results are expressed as median ± range **(F)**, the bar shows median levels **(G)**. **(H)** RNA *in situ* hybridization (RNAScope) staining of HDV-1cc (green) and HDV-3cc AG HDV RNA (red) in HDV-3cc/HDV-1cc sequential super-infected (**left**) and HDV-1cc control mice (**right**) at the end of the experiment. Nuclei are stained with dapi (blue).

We also investigated whether mice already chronically infected with HBV and HDV could be super-infected by another HDV genotype. We used stably HBV-infected USG mice and sequentially super-infected them with HDV-1cc for 9 weeks (first HDV super-infection) and then subsequently with HDV-3cc for additional 9 weeks (second HDV super-infection) (*n* = 2) (Figure 5A). After infection with HDV-1cc, mice developed viremia levels of around 1 × 10^8^ HDV-1 RNA copies/ml, which remained stable after super-infection with the second HDV strain (HDV-3cc) (Figure 5B). Surprisingly, genotype specific qRT-PCR showed that serum HDV-3cc RNA levels remained around the lower limit of detection (LLoD = 1 × 10^3^ copies/ml) for the entire observation time (Figure 5B). In contrast, control mice that had been infected only with HBV and super-infected with HDV-3cc (*n* = 2), developed as expected HDV-3cc viremia up to levels of 6 × 10^7^ copies/ml within 9 weeks (Figure 5B). In line with the HDV viremia, intrahepatic HDV-3cc RNA levels were clearly lower or below quantitative detection in mice that were previously infected with HDV-1cc, compared to mice that were only super-infected with HDV-3cc (Figure 5C). Using RNA *in situ* hybridization, we detected HDV-1cc AG RNA in approximately 20% of human hepatocytes at the end of experiment, while we were not able to detect HDV-3cc AG RNA-positive human hepatocytes in livers of these mice (Figure 5D). HBV-infected USG mice, which were super-infected with HDV-3cc alone, showed the presence of HDV-3cc antigenomic RNA-positive liver cells (Figure 5D). Conclusively, after an infection with HDV-1cc was established in HBV-infected USG mice, a second super-infection with HDV-3cc appeared strongly impaired.

Similarly, predominance of the HDV genotype that infected mouse livers first was determined also when stable HBV-infected mice were first super-infected with HDV-3cc and 9 weeks later with HDV-1cc (*n* = 3) (Figures 5E–H). Even in these mice, serum and liver HDV-1cc RNA levels hardly exceeded the LLoD, while HDV-3cc viremia (HDV-3cc 1 × 10^7^ copies/ml) and intrahepatic levels remained at high levels throughout the course of the experiment (Figures 5F,G). As expected, a control mouse that was infected with HBV and HDV-1cc only, developed serum HDV-1cc RNA levels of 8 × 10^6^ copies/ml (Figure 5F). In line with the previous experimental setting, HDV-1cc antigenomic RNA-positive human hepatocytes could only be detected in mice that were infected with HBV and HDV-1cc, but not in mice that already displayed a productive HDV-3cc infection (Figure 5H).

**FIGURE 6:**
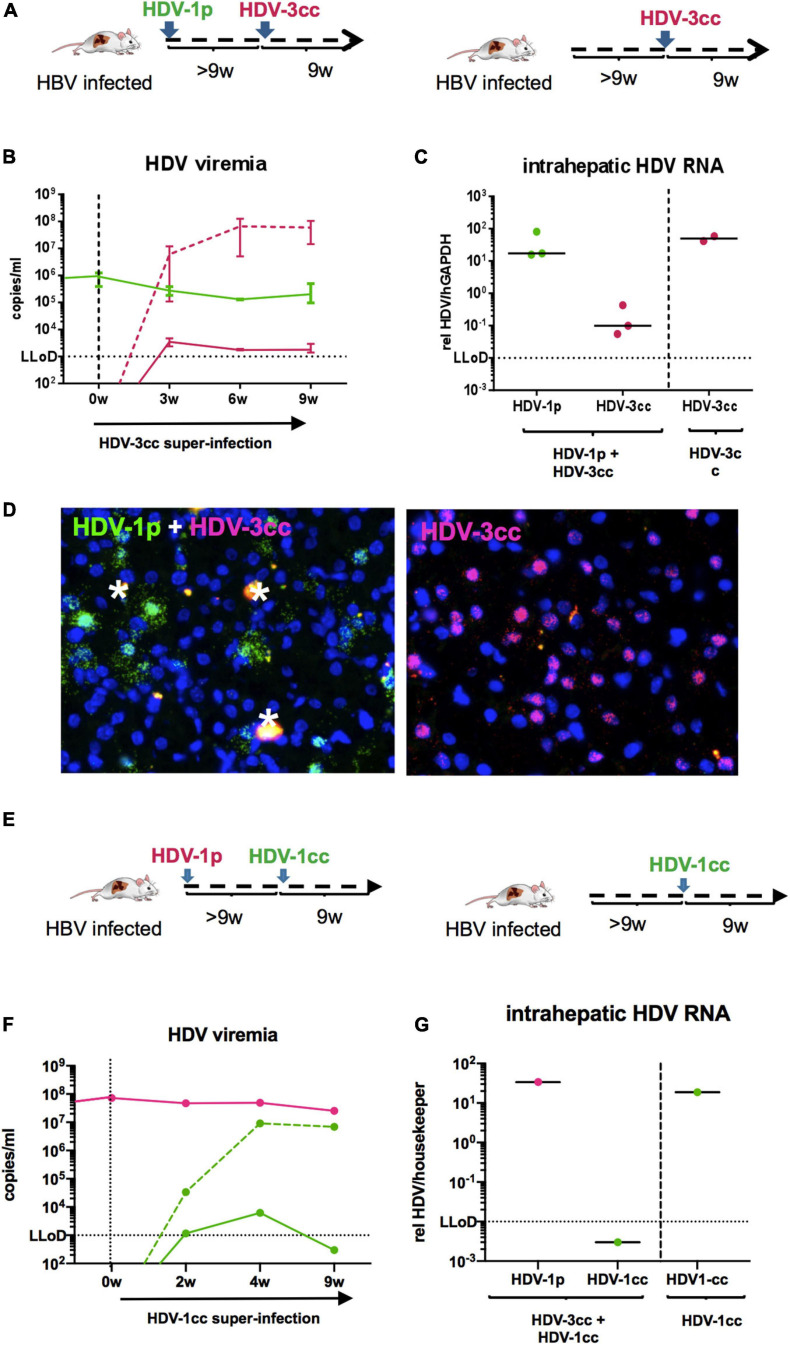
HDV in chronic HBV-infected mice sequentially super-infected with HDV-1p and HDV-3cc, or HDV-1p and HDV-1cc. **(A)** Experimental setting (first HDV-1p, then HDV-3cc; control mice were super-infected with HDV-3cc only). **(B,C)** qRT-PCR measurements (genotype specific HDV primers/probe for HDV-1 and HDV-3 were used) of serum HDV RNA (quantification with cell culture derived RNA standard) at different time-points **(B)** and liver HDV RNA (normalized to housekeeping gene hGAPDH) at the end of experiment **(C)**. HDV-1p RNA levels are depicted in green (line or dots) and HDV-3cc RNA levels in red (line or dots). The dashed red line shows HDV viremia in control mice (HDV-3cc only). Results are expressed as median ± range **(B)**, the bar shows median levels **(C)**. **(D)** RNA *in situ* hybridization (RNAScope) staining of HDV-1p (green) and HDV-3cc AG HDV RNA (red) in HDV-1p/HDV-3cc sequential super-infected (**left**) and HDV-3cc control mice (**right**) at the end of the experiment. Nuclei are stained with dapi (blue). Phagocytic remnants have an autofluorescence and are marked with a white star. **(E)** Experimental setting (first HDV-1p, then HDV-1cc; control mouse was super-infected with HDV-1cc only). **(F,G)** qRT-PCR measurements (strain specific HDV primers/probe for HDV-1cc and HDV-1p were used) of serum HDV RNA (quantification with cell culture derived RNA standard) at different time-points **(F)** and liver HDV RNA (normalized to housekeeping gene hGAPDH) at the end of experiment **(G)**. HDV-1p RNA levels are depicted in red (line or dots) and HDV-1cc RNA levels in green (line or dots). The dashed green line shows HDV viremia in control mouse (HDV-1cc only).

To exclude that the observed viral interference of HDV-1cc and HDV-3cc occurs exclusively among these two particular HDV strains, we also used stably HBV-infected mice and super-infected them first with a patient derived HDV-1 strain (HDV-1p) and then with HDV-3cc (*n* = 3) (Figure 6A). Even in this sequential super-infection setting, the presence of HDV-1p (serum HDV-1p 2 × 10^5^ copies/ml) substantially hindered the establishment of HDV-3cc infection, as demonstrated by the low levels (around the LLoD) of HDV-3cc RNA in serum and liver (Figures 6B,C) and by the absence of HDV-3cc antigenomic RNA-positive human hepatocytes in HBV/HDV-1p/HDV-3cc super-infected mice (RNA *in situ* hybridization, Figure 6D). HBV-infected control animals, which were super-infected with HDV-3cc (*n* = 2) only, developed HDV-3cc RNA viremia levels of 6 × 10^7^ copies/ml (Figure 6B) and showed the presence of HDV-3cc antigenomic RNA-positive liver cells (Figure 6D).

Finally, and in an attempt to investigate whether viral interference mechanisms may hinder the establishment of productive super-infections also among HDV strains of the same genotype, one HBV/HDV-1p infected humanized mouse was super-infected with HDV-1cc (Figure 6E). Interestingly, even after 9 weeks of super-infection with the second HDV-1 isolate no serum and liver HDV-1cc RNAs were detected, while HDV-1p viremia remained unchanged, whereas one mouse that was infected in parallel with HBV and HDV-1cc only, - as expected - developed high HDV-1cc serum RNA levels (Figures 6F,G).

Overall, our sequential super-infection experiments in USG mice revealed that an already established HDV infection strongly impairs the establishment of a productive subsequent HDV infection not only with a genetically distant genotype, but also with a similar isolate.

## Discussion

Hepatitis D Virus is classified into eight different genotypes with a sequence divergence from 15% up to 40% according to partial or full-length genome sequencing (Deny, 2006; Le Gal et al., 2017). Despite the presence of conserved nucleotides, amino acid motifs and positions across all genotypes, indicating the existence of conservatory constraints both at genomic and protein level, inter-genotype differences are also linked to distinct geographical distributions, and different treatment responses and pathogenicity. HDV-1 infection is distributed around the world, shows diverse clinical outcomes and was studied the most *in vitro*, *in vivo*, and in patients. HDV-3 is geographically isolated in the Amazonas region, is associated with the most severe disease outcomes among all HDV genotypes (Casey et al., 1996), but seems to respond better to treatment with PEGylated interferon alpha than HDV-1 (Borzacov et al., 2016). Nevertheless, only very few HDV strains have been studied *in vivo* to date.

Taking advantage of the availability of a commonly used HDV-1 clone (Kuo et al., 1988, 1989) and an HDV-3 clone isolated and generated from an infected Peruvian man with acute severe hepatitis (Casey et al., 1993), in this study we first investigated virological differences between HDV-1 and HDV-3 in primary human hepatocytes co-infected with HBV *in vivo*. By using USG mice that were repopulated with human hepatocytes we observed that HDV-3 establishes higher rates of HDV viremia and intrahepatic HDV RNA levels than this HDV-1 strain in HBV-infected chimeric mice. Despite the use of inocula containing comparable levels of HDV genome equivalents, immunofluorescence staining and RNA *in situ* hybridization revealed that around 70% of human hepatocytes were infected with HDV-3, while only 20% of human cells were HDV-1-positive. In the absence of HBV, HDV-1, and HDV-3 were able to initially infect comparable amounts of human cells *in vitro* (data not shown) and *in vivo* (Giersch et al., 2021), indicating that HDV1 and HDV3 mainly differ in their spreading and assembly capacities. HDV has been observed to suppress HBV replication (Niro et al., 2001; Giersch and Dandri, 2015) and indeed the higher infection levels of HDV-3 determined in this study were accompanied by a stronger suppression of serum and intrahepatic HBV markers compared to HBV/HDV-1 infection.

An *in vitro* study suggested that the severity of hepatic inflammation might be associated with distinct efficacies of HDV genotypes to generate L-HDAg, package and finally secrete viral particles (Hsu et al., 2002). The authors observed that HDV-2 secretes fewer viral particles than HDV-1, which may reduce the extent of liver infection and result in less severe hepatic inflammation (Hsu et al., 2002). While genome sequencing in our study revealed that HDV editing at the amber/W site and thus the production of genomes encoding for the L-HDAg was comparatively lower in HDV-3 infection, the amounts of S- and L-HDAg determined by western blot analysis were similar in HDV-1- and HDV-3-infected animals. However, we observed a higher intracellular productivity of HDV-3, which may explain the faster spreading kinetics and increased numbers of infected cells achieved in the same time frame in comparison to HDV-1 and therefore contribute to the more severe clinical course of HDV-3 infections. Moreover, the degree of apoptosis and liver injury appeared low and comparable with both strains, while the induction of some human specific interferon stimulated genes (hISG15, hCXCL10) tended to be slightly higher in mice harboring HDV-3, suggesting that an enhancement of the intrinsic innate responses may contribute to augment the severity of HDV-3-associated liver disease. However, these mice lack B, T, and NK cells and it is also plausible that the severity of HDV-3 in patients may rather be caused by responses of the adaptive immune system or by so far unknown genotype specific events.

Secondly, we investigated whether HDV-1 and HDV-3 could co-infect the same liver, a setting prone to genetic recombination. To date, co-infections of HDV-1 and HDV-2 were observed *in vitro* and in patients (Wu et al., 1999; Wang and Chao, 2005; Sy et al., 2015), but not between HDV-1 and HDV-3 (Cicero et al., 2016).

Here, we observed for the first time that co-infections of HDV-1 and HDV-3 are possible in human liver chimeric mice already infected with HBV. However, one strain (HDV-3) became dominant within 10 weeks after infection, while viremia and intrahepatic viral loads of the other HDV-1 strain used in these experiments were clearly decreased. Interestingly, even at an early time-point of HDV-1/HDV-3 co-infection, when both HDV genotypes were clearly detected in the liver, we could rarely find human hepatocytes harboring HDV-1 and HDV-3 AG RNA at the same time, indicating that the two genotypes are not able to replicate efficiently within the same cell. Furthermore, we inoculated chronic HBV-infected mice first with HDV-1 and several weeks later with HDV-3 (and vice versa) and demonstrated that when an infection with one HDV genotype is already established in humanized livers, the super-infection with another genotype was strongly impaired. Remarkably, also when one mouse was stably infected with one HDV-1 isolate, a super-infection with another HDV-1 strain could not be established. Taken together, these results indicate the existence of a strong viral inter- and intragenotypic interference and may allow HDV to evade excessive recombination by incoming, divergent viruses, which is believed to occur through RNA template switching of the host RNA polymerase during replication of G HDV RNA (Chao, 2007; Lin et al., 2015). Recombination of different genotypes can occur when at least two viral genomes co-infect the same host cell and is observed among RNA viruses such as hepatitis C virus (HCV), influenza A virus and human immunodeficiency virus (HIV) (Perez-Losada et al., 2015). In general, inter-genomic recombination is more likely to occur in plus-strand viruses (McVean et al., 2002), but was also described for HDV-1 and HDV-2 *in vitro* and in patients (Wang and Chao, 2005; Sy et al., 2015). Lin et al. (2015) observed *in vitro* that intra-genotypic recombination rates (between two different HDV-1 isolates) were higher compared to recombination rates that were obtained between different genotypes (HDV-1 and HDV-2 or HDV-4) and proposed that a decreased genetic distance favors HDV recombination frequency. Although, in this study simultaneous super-infection with two different HDV-1 isolates, led to comparable levels of both HDV-1 strains in serum and liver, NGS analysis of HDV1/3 and HDV1/1 infected mice failed to detect inter- or intra-genotypic recombination within the observed time. These results indicate that simultaneous replication of different HDV genotypes and strains in the same human hepatocyte do not occur frequently *in vivo*, not even in the absence of an adaptive immune system, which could affect HDV spreading capacities. HDV recombination events described in patients may require a longer-term coexistence in the same liver and perhaps even immune mediated selective pressures favoring co-infection and recombination events.

Casey and Gerin (1998) performed co-transfection experiments using HuH7 cells and observed that expression of HDV-3 S-HDAg strongly inhibited HDV-1 RNA replication, while transfection of HDV-1 S-HDAg resulted in a two- to threefold inhibition of HDV-3 RNA replication. The natural function of the L-HDAg is to suppress HDV replication (and support virus assembly) and interestingly, the L-HDAg of HDV-3 was shown to decrease not only HDV-3 but also HDV-1 replication. Vice versa, the L-HDAg of HDV-1 was also able to suppress both HDV-1 and HDV-3 replication, even though the sequence of the C-terminal extension of the HDAg responsible for this inhibitory activity (Chao et al., 1990) is highly different between the two genotypes (Casey and Gerin, 1998). Therefore, it is conceivable that also in our study the presence of S- and L-HDAgs from HDV-1 hindered the establishment and in particular the replication capacities of HDV-3 (and vice versa) in the same human hepatocyte. Future studies are needed to proof whether the inability of HDAg to support replication of another more distant HDV genotype is indeed the main factor responsible for the observed interference between HDV-1 and HDV-3 *in vivo*.

It is striking that even though only about 20% of human hepatocytes were infected with HDV-1, a sequential super-infection with HDV-3 was not achievable in the “remaining” 80% of the human hepatocytes. We showed that the induction of innate immune responses is significantly higher in HBV/HDV co-infected than in HBV mono-infected human liver chimeric mice (Giersch et al., 2015). Interestingly, this study revealed that the expression of human ISGs (i.e., hISG15, hMxA) was not only elevated in HDV-infected cells but also in the surrounding HDV-negative hepatocytes, suggesting that HDV infection induces a broad antiviral state of the host, which may also hinder super-infection with another HDV genotype and even with another HDV isolate of the same genotype. This hypothesis is in line with a publication by Han et al. (2011) who showed that the host antiviral response induced by interferon treatment in primary human hepatocytes, was largely mediated at the level of HDV entry. The same study also observed that hepatocytes already infected with HDV were resistant to a second infection with vesicular stomatitis virus (VSV).

Overall, our experiments in human liver chimeric mice clearly demonstrated that simultaneous infection with the two most distinct HDV genotypes 1 and 3 is possible within the same liver at least for a short period of time, while replication of two different HDV isolates within the same human hepatocyte almost never occurred in the setting of co- or sequential super-infection. The observed viral interference between HDV genotypes would hamper their co-existence in the same individual and thus their recombination. Such co-infection interferences might also contribute to the geographical isolation of HDV-3 and to a further divergent development of these already genetically distinct genotypes.

## Data Availability Statement

The datasets presented in this study can be found in online repositories. The names of the repository/repositories and accession number(s) can be found below: EBI, PRJEB44735.

## Ethics Statement

All animal experiments were conducted in accordance with the European Communities Council Directive (86/609/EEC) and were approved by the City of Hamburg, Germany. Written informed consent was obtained from the owners for the participation of their animals in this study.

## Author Contributions

ML and MD initiated and supervised the study. ML, MD, KG, and LH designed experiments. LA and TV generated chimeric mice. KG, LH, and AV performed analyses and generated data. JH and NF performed NGS analysis. CS provided infectious HDV-1 and HDV-3 cell culture supernatant. JC provided the HDV-3 pCMV3-Peru-1.2 plasmid. KG, LH, and MD wrote the manuscript. LA, TV, AV, ML, CS, and JC discussed the data and corrected the manuscript. All authors contributed to the article and approved the submitted version.

## Conflict of Interest

The authors declare that the research was conducted in the absence of any commercial or financial relationships that could be construed as a potential conflict of interest.

## Supplementary Material

Supplementary Figure 1 HDV RNA amplification and standard curves. HDV RNA was extracted from HuH7 cell culture supernatant, which contained either HDV-1cc, HDV-1p, or HDV-3cc virions, as described in Materials and Methods, and HDV RNA standard curves using primers and probes specific for HDV-1 (all isolates), HDV-1cc, HDV1-p, or HDV-3 were prepared in 1:5 dilutions.

Supplementary Figure 2 G/AG HDV RNAs, genome sequence and HDAgs in mice co-infected with HBV and HDV-1cc or HDV-3cc. **(A)** qRT-PCR measurements (dynabead based assay, see Materials and Methods) of liver G and AG HDV RNA (normalized to housekeeping gene hGAPDH) in HBV/HDV-1cc (green) and HBV/HDV-3cc (red) co-infected mice 9 weeks post infection. The bar shows median levels. **(B)** Sanger sequencing of genomic HDV RNA in serum of HBV/HDV-1cc and HBV/HDV-3cc co-infected mice 9 weeks post infection. Displayed is the HDV amber/W site. The sequence ATG represents a stop codon and thus encodes for the S-HDAg, while RNA editing leads to the sequence ACC, which encodes for the L-HDAg. **(C)** Western blot analysis of intrahepatic S- (24 kDa) and L-HDAg (27 kDa) in two HBV/HDV-3 (left two lanes) and HBV/HDV-1 (right two lanes) infected mice.

Supplementary Figure 3 Cell death markers in mice co-infected with HBV and HDV-1cc or HDV-3cc. **(A)** qRT-PCR measurement of intrahepatic human caspase 8 (normalized to median of housekeeping genes hGAPDH and hRPL30) in HBV/HDV-1cc and HBV/HDV-3cc co-infected mice 9 weeks post infection compared to uninfected mice. The bar shows median levels. **(B)** Human serum albumin (ELISA) in HBV/HDV-1cc and HBV/HDV-3cc co-infected mice at different time-points of the experiment.

Supplementary Figure 4 Correlation of liver HDV RNA and hISG mRNA. qRT-PCR measurements of liver HDV RNA (normalized to housekeeping gene hGAPDH) and hISG15 or hCXCL10 (normalized to median of housekeeping genes hGAPDH and hRPL30) in HBV/HDV-3cc (red dots) and HBV/HDV-1cc infected mice (green dots) 9 weeks post infection. Correlations between HDV RNA and hISG15 or hCXCL10 show a *p*-value of *p* = 0.0323* (spearman r = 0.63) and *p* = 0.0591 (spearman r = 0.57), respectively, when HBV/HDV-1cc and HBV/HDV-3cc infected mice are analyzed together.

Supplementary Figure 5 Intrahepatic HLA ABC expression. Immunofluorescence staining of human HLA ABC (red), HBcAg (green), HDAg (turquoise) and overlays of HLA ABC with HBcAg or with HBcAg and HDAg in HBV/HDV-1cc (left) and HBV/HDV-3cc (right) co-infected mice at the end of the experiment. Nuclei are stained with Hoechst 33258 (blue).

Supplementary Figure 6 HBV in chronic HBV-infected mice simultaneously super-infected with HDV-1cc and HDV-3cc. qRT-PCR measurements of serum HBV DNA (quantification with plasmid standard) **(A)** and liver pregenomic HBV RNA (normalized to housekeeping gene hGAPDH) **(B)** in chronic HBV-infected mice simultaneously super-infected with HDV-1cc and HDV-3cc (red line or dots) compared to stable HBV mono-infected mice (black line or dots) at indicated time-points post HDV super-infection. Results are expressed as median ± range **(A)**, the bar shows median levels **(B)**.

## Abbreviations

AG: antigenomic
ALT: alanine aminotransferase
cc: cell culture derived
cccDNA: covalently closed circular DNA
CK18: cytokeratin 18
CXCL10: C-X -C motif chemokine ligand 10
G: genomic
GAPDH: glyceraldehyde-3 phosphate dehydrogenase
HBsAg: hepatitis B surface antigen
HBV: hepatitis B virus
HDAg: hepatitis delta antigen (S, small, L, large)
HCV: hepatitis C virus
HDV: hepatitis delta virus
HIV: human immunodeficiency virus
HLA: human leukocyte antigen
HSA: human serum albumin
ISGs: interferon stimulated genes
LLoD: lower limit of detection
MxA: Myxovirus resistance gene A
NK: natural killer
NTCP: natrium taurocholate polypeptide
p: patient derived
OAS1: 2′-5′-oligoadenylate synthetase 1
pgRNA: pregenomic RNA
qRT-PCR: quantitative real time polymerase chain reaction
SCID: severe combined immunodeficiency
uPA: urokinase plasminogen activator
USG: uPA/SCID/beige/IL2RG^–/–^.

## References

[B1] Allweiss L. Gass S. Giersch K. Groth A. Kah J. Volz T. (2016). Human liver chimeric mice as a new model of chronic hepatitis E virus infection and preclinical drug evaluation. *J. Hepatol.* 64 1033–1040. 10.1016/j.jhep.2016.01.011 26805671

[B2] Bankevich A. Nurk S. Antipov D. Gurevich A. A. Dvorkin M. Kulikov A. S. (2012). SPAdes: a new genome assembly algorithm and its applications to single-cell sequencing. *J. Comput. Biol.* 19 455–477. 10.1089/cmb.2012.0021 22506599 PMC3342519

[B3] Bolger A. M. Lohse M. Usadel B. (2014). Trimmomatic: a flexible trimmer for Illumina sequence data. *Bioinformatics* 30 2114–2120. 10.1093/bioinformatics/btu170 24695404 PMC4103590

[B4] Borzacov L. M. de Figueiredo Nicolete L. D. Souza L. F. Dos Santos A. O. Vieira D. S. Salcedo J. M. (2016). Treatment of hepatitis delta virus genotype 3 infection with peg-interferon and entecavir. *Int. J. Infect. Dis.* 46 82–88. 10.1016/j.ijid.2016.03.017 27005283

[B5] Botelho-Souza L. F. Vasconcelos M. P. A. Dos Santos A. O. Salcedo J. M. V. Vieira D. S. (2017). Hepatitis delta: virological and clinical aspects. *Virol. J.* 14:177. 10.1186/s12985-017-0845-y PMC5597996 28903779

[B6] Cappy P. Lucas Q. Kankarafou N. Sureau C. Laperche S. (2020). No evidence of HCV-assisted HDV propagation in a large cohort of hepatitis C positive blood donors. *J. Infect. Dis.* 223 1376–1380. 10.1093/infdis/jiaa517 32804999

[B7] Casey J. L. Gerin J. L. (1998). Genotype-specific complementation of hepatitis delta virus RNA replication by hepatitis delta antigen. *J. Virol.* 72 2806–2814. 10.1128/jvi.72.4.2806-2814.1998 9525600 PMC109725

[B8] Casey J. L. Brown T. L. Colan E. J. Wignall F. S. Gerin J. L. (1993). A genotype of hepatitis D virus that occurs in northern South America. *Proc. Natl. Acad. Sci. U.S.A.* 90 9016–9020. 10.1073/pnas.90.19.9016 8415646 PMC47492

[B9] Casey J. L. Niro G. A. Engle R. E. Vega A. Gomez H. McCarthy M. (1996). Hepatitis B virus (HBV)/hepatitis D virus (HDV) coinfection in outbreaks of acute hepatitis in the Peruvian Amazon basin: the roles of HDV genotype III and HBV genotype F. *J. Infect. Dis.* 174 920–926. 10.1093/infdis/174.5.920 8896491

[B10] Chang F. L. Chen P. J. Tu S. J. Wang C. J. Chen D. S. (1991). The large form of hepatitis delta antigen is crucial for assembly of hepatitis delta virus. *Proc. Natl. Acad. Sci. U.S.A.* 88 8490–8494. 10.1073/pnas.88.19.8490 1924308 PMC52534

[B11] Chao M. (2007). RNA recombination in hepatitis delta virus: implications regarding the abilities of mammalian RNA polymerases. *Virus Res.* 127 208–215. 10.1016/j.virusres.2007.01.003 17296240

[B12] Chao M. Hsieh S. Y. Taylor J. (1990). Role of two forms of hepatitis delta virus antigen: evidence for a mechanism of self-limiting genome replication. *J. Virol.* 64 5066–5069. 10.1128/jvi.64.10.5066-5069.1990 2398535 PMC247998

[B13] Chemin I. Pujol F. H. Scholtes C. Loureiro C. L. Amirache F. Levrero M. (2020). Preliminary evidence for hepatitis delta virus exposure in patients who are apparently not infected with hepatitis B virus. *Hepatology.* 73 861–864. 10.1002/hep.31453 32628280 PMC7898870

[B14] Chen H. Y. Shen D. T. Ji D. Z. Han P. C. Zhang W. M. Ma J. F. (2018). Prevalence and burden of hepatitis D virus infection in the global population: a systematic review and meta-analysis. *Gut.* 68 512–521. 10.1136/gutjnl-2018-316601 30228220

[B15] Cicero M. F. Pena N. M. Santana L. C. Arnold R. Azevedo R. G. Leal E. S. (2016). Is hepatitis delta infections important in Brazil? *BMC Infect. Dis.* 16:525. 10.1186/s12879-016-1856-9 27686363 PMC5041555

[B16] Dandri M. Murray J. M. Lutgehetmann M. Volz T. Lohse A. W. Petersen J. (2008). Virion half-life in chronic hepatitis B infection is strongly correlated with levels of viremia. *Hepatology* 48 1079–1086. 10.1002/hep.22469 18697217

[B17] Delfino C. M. Cerrudo C. S. Biglione M. Oubina J. R. Ghiringhelli P. D. Mathet V. L. (2018). A comprehensive bioinformatic analysis of hepatitis D virus full-length genomes. *J. Viral Hepat.* 25 860–869. 10.1111/jvh.12876 29406571

[B18] Deny P. (2006). Hepatitis delta virus genetic variability: from genotypes I, II, III to eight major clades? *Curr. Top. Microbiol. Immunol.* 307 151–171. 10.1007/3-540-29802-9_8 16903225

[B19] Ferns R. B. Nastouli E. Garson J. A. (2011). Quantitation of hepatitis delta virus using a single-step internally controlled real-time RT-qPCR and a full-length genomic RNA calibration standard. *J. Virol. Methods* 179 189–194. 10.1016/j.jviromet.2011.11.001 22108292

[B20] Freitas N. Cunha C. Menne S. Gudima S. O. (2014). Envelope proteins derived from naturally integrated hepatitis B virus DNA support assembly and release of infectious hepatitis delta virus particles. *J. Virol.* 88 5742–5754. 10.1128/jvi.00430-14 24623409 PMC4019103

[B21] Giersch K. Allweiss L. Volz T. Helbig M. Bierwolf J. Lohse A. W. (2015). Hepatitis Delta co-infection in humanized mice leads to pronounced induction of innate immune responses in comparison to HBV mono-infection. *J. Hepatol.* 63 346–353. 10.1016/j.jhep.2015.03.011 25795587

[B22] Giersch K. Dandri M. (2015). Hepatitis B and Delta Virus: advances on studies about interactions between the two viruses and the infected hepatocyte. *J. Clin. Transl. Hepatol.* 3 220–229. 10.14218/jcth.2015.00018 26623269 PMC4663204

[B23] Giersch K. Bhadra O. D. Volz T. Allweiss L. Riecken K. Fehse B. (2019). Hepatitis delta virus persists during liver regeneration and is amplified through cell division both in vitro and in vivo. *Gut* 68 150–157. 10.1136/gutjnl-2017-314713 29217749

[B24] Giersch K. Hermanussen L. Volz T. Kah J. Allweiss L. Casey J. (2021). Murine hepatocytes do not support persistence of Hepatitis D virus mono-infection in vivo. *Liver Int.* 41 410–419. 10.1111/liv.14677 32997847

[B25] Gudima S. He Y. Meier A. Chang J. Chen R. Jarnik M. (2007). Assembly of hepatitis delta virus: particle characterization, including the ability to infect primary human hepatocytes. *J. Virol.* 81 3608–3617. 10.1128/jvi.02277-06 17229685 PMC1866043

[B26] Han Z. Nogusa S. Nicolas E. Balachandran S. Taylor J. (2011). Interferon impedes an early step of hepatitis delta virus infection. *PLoS One* 6:e22415. 10.1371/journal.pone.0022415 21811602 PMC3139649

[B27] Hsu S. C. Syu W. J. Sheen I. J. Liu H. T. Jeng K. S. Wu J. C. (2002). Varied assembly and RNA editing efficiencies between genotypes I and II hepatitis D virus and their implications. *Hepatology* 35 665–672. 10.1053/jhep.2002.31777 11870382

[B28] Ivaniushina V. Radjef N. Alexeeva M. Gault E. Semenov S. Salhi M. (2001). Hepatitis delta virus genotypes I and II cocirculate in an endemic area of Yakutia, Russia. *J. Gen. Virol.* 82 2709–2718. 10.1099/0022-1317-82-11-2709 11602783

[B29] Koh C. Heller T. Glenn J. S. (2019). Pathogenesis of and new therapies for hepatitis D. *Gastroenterology* 156 461–476.e1. 30342879 10.1053/j.gastro.2018.09.058 PMC6340762

[B30] Kuo M. Y. Chao M. Taylor J. (1989). Initiation of replication of the human hepatitis delta virus genome from cloned DNA: role of delta antigen. *J. Virol.* 63 1945–1950. 10.1128/jvi.63.5.1945-1950.1989 2649689 PMC250607

[B31] Kuo M. Y. Goldberg J. Coates L. Mason W. Gerin J. Taylor J. (1988). Molecular cloning of hepatitis delta virus RNA from an infected woodchuck liver: sequence, structure, and applications. *J. Virol.* 62 1855–1861. 10.1128/jvi.62.6.1855-1861.1988 3367426 PMC253266

[B32] Lai M. M. (2005). RNA replication without RNA-dependent RNA polymerase: surprises from hepatitis delta virus. *J. Virol.* 79 7951–7958. 10.1128/jvi.79.13.7951-7958.2005 15956541 PMC1143735

[B33] Langmead B. Salzberg S. L. (2012). Fast gapped-read alignment with Bowtie 2. *Nat. Methods* 9 357–359. 10.1038/nmeth.1923 22388286 PMC3322381

[B34] Le Gal F. Brichler S. Drugan T. Alloui C. Roulot D. Pawlotsky J. M. (2017). Genetic diversity and worldwide distribution of the deltavirus genus: a study of 2,152 clinical strains. *Hepatology* 66 1826–1841. 10.1002/hep.29574 28992360

[B35] Le Gal F. Gault E. Ripault M. P. Serpaggi J. Trinchet J. C. Gordien E. (2006). Eighth major clade for hepatitis delta virus. *Emerg. Infect. Dis.* 12 1447–1450. 10.3201/eid1209.060112 17073101 PMC3294742

[B36] Lin C. C. Yang Z. W. Iang S. B. Chao M. (2015). Reduced genetic distance and high replication levels increase the RNA recombination rate of hepatitis delta virus. *Virus Res.* 195 79–85. 10.1016/j.virusres.2014.08.011 25172581

[B37] Lutgehetmann M. Mancke L. V. Volz T. Helbig M. Allweiss L. Bornscheuer T. (2012). Humanized chimeric uPA mouse model for the study of hepatitis B and D virus interactions and preclinical drug evaluation. *Hepatology* 55 685–694. 10.1002/hep.24758 22031488

[B38] Magoc T. Salzberg S. L. (2011). FLASH: fast length adjustment of short reads to improve genome assemblies. *Bioinformatics* 27 2957–2963. 10.1093/bioinformatics/btr507 21903629 PMC3198573

[B39] Malmstrom S. Larsson S. B. Hannoun C. Lindh M. (2012). Hepatitis B viral DNA decline at loss of HBeAg is mainly explained by reduced cccDNA load–down-regulated transcription of PgRNA has limited impact. *PLoS One* 7:e36349. 10.1371/journal.pone.0036349 22911677 PMC3401194

[B40] McVean G. Awadalla P. Fearnhead P. (2002). A coalescent-based method for detecting and estimating recombination from gene sequences. *Genetics* 160 1231–1241. 10.1093/genetics/160.3.1231 11901136 PMC1462015

[B41] Mederacke I. Bremer B. Heidrich B. Kirschner J. Deterding K. Bock T. (2010). Establishment of a novel quantitative hepatitis D virus (HDV) RNA assay using the Cobas TaqMan platform to study HDV RNA kinetics. *J. Clin. Microbiol.* 48 2022–2029. 10.1128/jcm.00084-10 20351206 PMC2884474

[B42] Niro G. A. Gravinese E. Martini E. Garrubba M. Facciorusso D. Conoscitore P. (2001). Clearance of hepatitis B surface antigen in chronic carriers of hepatitis delta antibodies. *Liver* 21 254–259. 10.1034/j.1600-0676.2001.021004254.x 11454188

[B43] Perez-Losada M. Arenas M. Galan J. C. Palero F. Gonzalez-Candelas F. (2015). Recombination in viruses: mechanisms, methods of study, and evolutionary consequences. *Infect. Genet. Evol.* 30 296–307. 10.1016/j.meegid.2014.12.022 25541518 PMC7106159

[B44] Perez-Vargas J. Amirache F. Boson B. Mialon C. Freitas N. Sureau C. (2019). Enveloped viruses distinct from HBV induce dissemination of hepatitis D virus in vivo. *Nat. Commun.* 10:2098. 10.1038/s41467-019-10117-z PMC6506506 31068585

[B45] Pfluger L. S. Schulze Zur Wiesch J. Polywka S. Lutgehetmann M. (2021). Hepatitis delta virus propagation enabled by hepatitis C virus-Scientifically intriguing, but is it relevant to clinical practice? *J. Viral Hepat.* 28 213–216. 10.1111/jvh.13385 32852870

[B46] Rizzetto M. Hoyer B. Canese M. G. Shih J. W. Purcell R. H. Gerin J. L. (1980). delta Agent: association of delta antigen with hepatitis B surface antigen and RNA in serum of delta-infected chimpanzees. *Proc. Natl. Acad. Sci. U.S.A.* 77 6124–6128. 10.1073/pnas.77.10.6124 6934539 PMC350226

[B47] Sy B. T. Nguyen H. M. Toan N. L. Song L. H. Tong H. V. Wolboldt C. (2015). Identification of a natural intergenotypic recombinant hepatitis delta virus genotype 1 and 2 in Vietnamese HBsAg-positive patients. *J. Viral Hepat.* 22 55–63. 10.1111/jvh.12228 24548489

[B48] Wang C. J. Chen P. J. Wu J. C. Patel D. Chen D. S. (1991). Small-form hepatitis B surface antigen is sufficient to help in the assembly of hepatitis delta virus-like particles. *J. Virol.* 65 6630–6636. 10.1128/jvi.65.12.6630-6636.1991 1658366 PMC250729

[B49] Wang K. S. Choo Q. L. Weiner A. J. Ou J. H. Najarian R. C. Thayer R. M. (1986). Structure, sequence and expression of the hepatitis delta (delta) viral genome. *Nature* 323 508–514. 10.1038/323508a0 3762705

[B50] Wang T. C. Chao M. (2005). RNA recombination of hepatitis delta virus in natural mixed-genotype infection and transfected cultured cells. *J. Virol.* 79 2221–2229. 10.1128/jvi.79.4.2221-2229.2005 15681424 PMC546541

[B51] Wu J. C. Huang I. A. Huang Y. H. Chen J. Y. Sheen I. J. (1999). Mixed genotypes infection with hepatitis D virus. *J. Med. Virol.* 57 64–67. 10.1002/(sici)1096-9071(199901)57:1<64::aid-jmv9>3.0.co;2-w 9890423

[B52] Yan H. Zhong G. Xu G. He W. Jing Z. Gao Z. (2012). Sodium taurocholate cotransporting polypeptide is a functional receptor for human hepatitis B and D virus. *Elife* 1:e00049. 10.7554/eLife.00049 PMC3485615 23150796

