# Supplementary figures and images for: Strong Replication Interference Between Hepatitis Delta Viruses in Human Liver Chimeric Mice
Source: Front Microbiol. 2021 Jul 8;12:671466. doi: 10.3389/fmicb.2021.671466 (PMC8297590; doi:10.3389/fmicb.2021.671466)

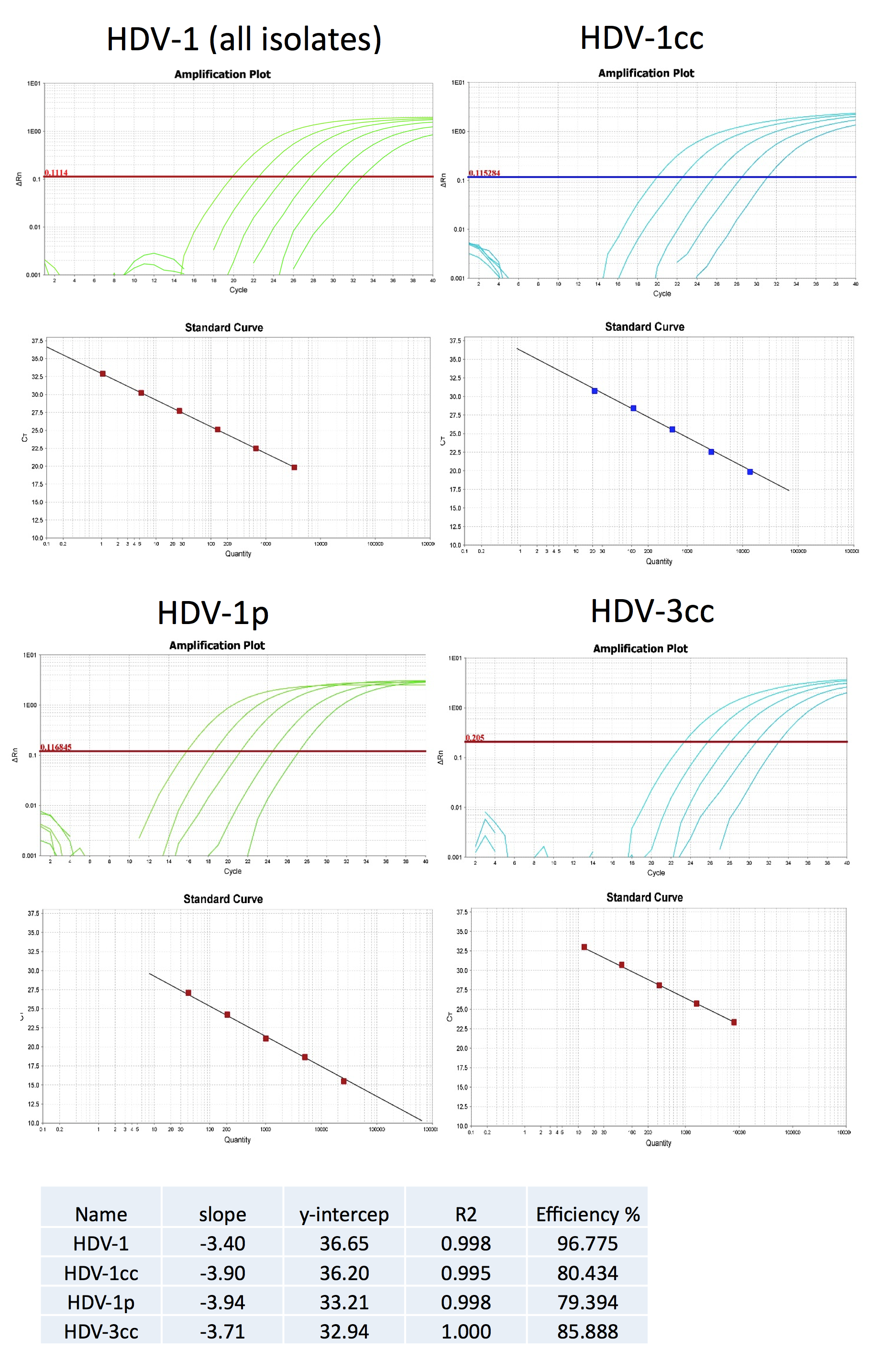

Supplement: Supplementary Figure 1 — HDV RNA amplification and standard curves. HDV RNA was extracted from HuH7 cell culture supernatant, which contained either HDV-1cc, HDV-1p, or HDV-3cc virions, as described in Materials and Methods, and HDV RNA standard curves using primers and probes specific for HDV-1 (all isolates), HDV-1cc, HDV1-p, or HDV-3 were prepared in 1:5 dilutions. [file Image_1.tiff]

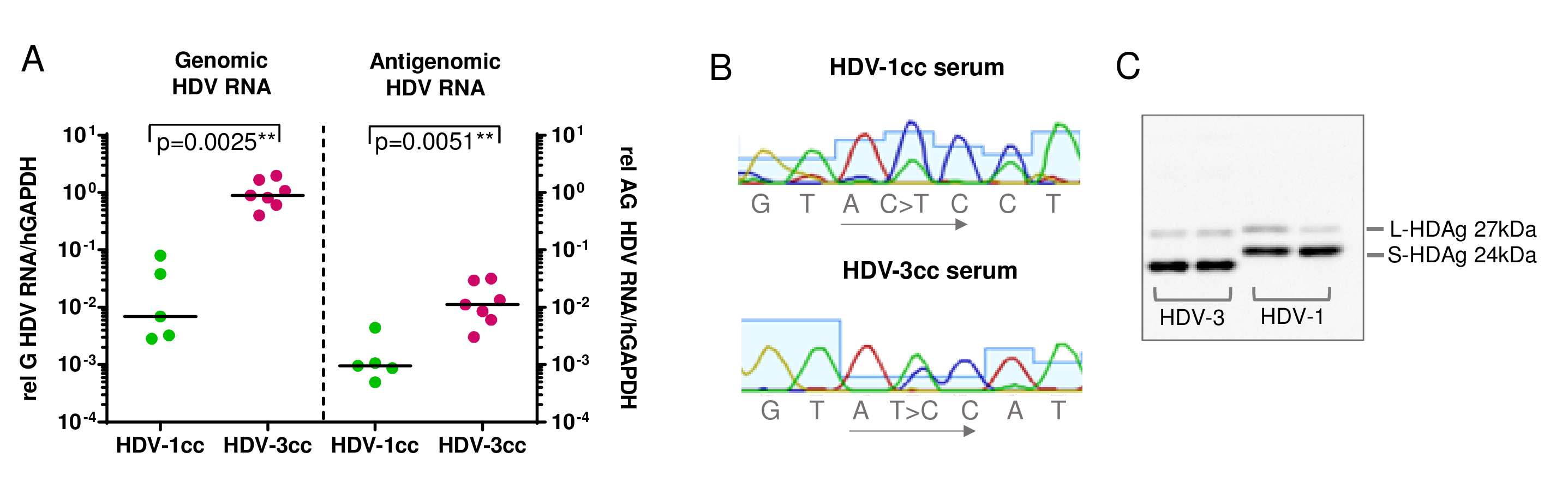

Supplement: Supplementary Figure 2 — G/AG HDV RNAs, genome sequence and HDAgs in mice co-infected with HBV and HDV-1cc or HDV-3cc. (A) qRT-PCR measurements (dynabead based assay, see Materials and Methods) of liver G and AG HDV RNA (normalized to housekeeping gene hGAPDH) in HBV/HDV-1cc (green) and HBV/HDV-3cc (red) co-infected mice 9 weeks post infection. The bar shows median levels. (B) Sanger sequencing of genomic HDV RNA in serum of HBV/HDV-1cc and HBV/HDV-3cc co-infected mice 9 weeks post infection. Displayed is the HDV amber/W site. The sequence ATG represents a stop codon and thus encodes for the S-HDAg, while RNA editing leads to the sequence ACC, which encodes for the L-HDAg. (C) Western blot analysis of intrahepatic S- (24 kDa) and L-HDAg (27 kDa) in two HBV/HDV-3 (left two lanes) and HBV/HDV-1 (right two lanes) infected mice. [file Image_2.tif]

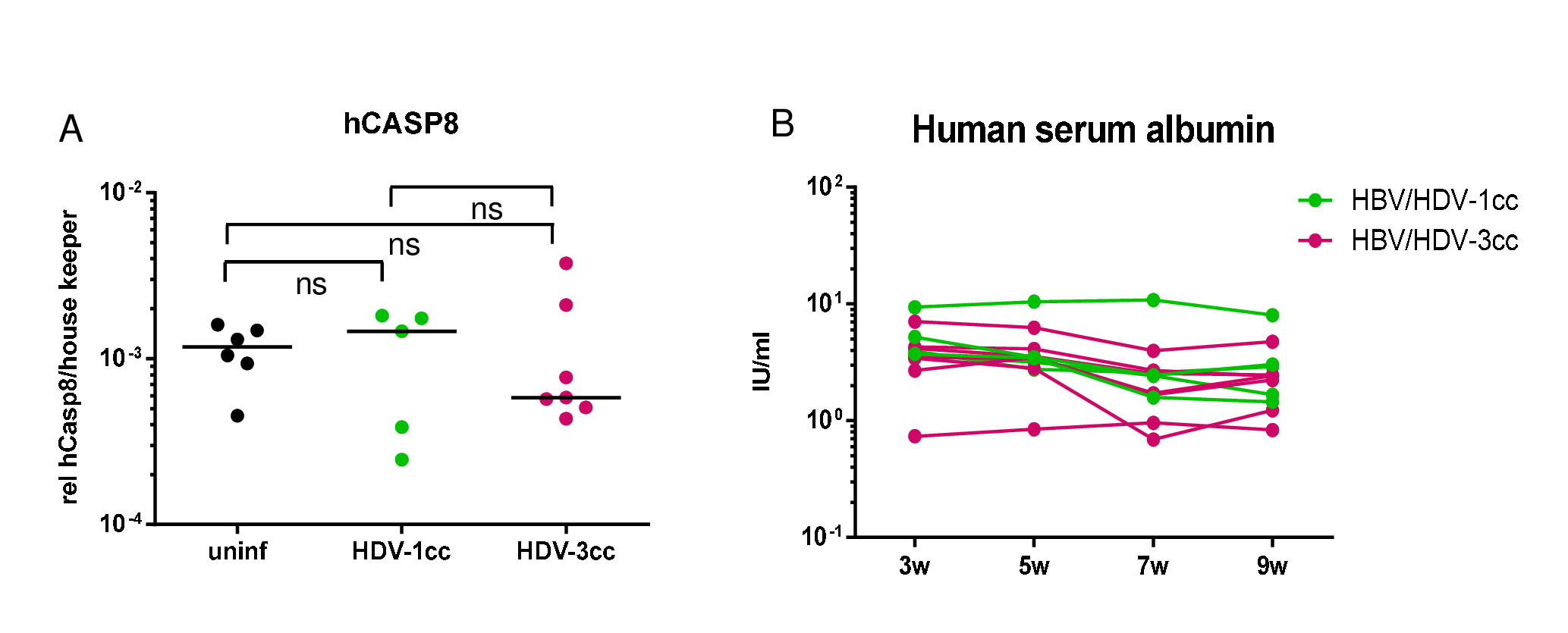

Supplement: Supplementary Figure 3 — Cell death markers in mice co-infected with HBV and HDV-1cc or HDV-3cc. (A) qRT-PCR measurement of intrahepatic human caspase 8 (normalized to median of housekeeping genes hGAPDH and hRPL30) in HBV/HDV-1cc and HBV/HDV-3cc co-infected mice 9 weeks post infection compared to uninfected mice. The bar shows median levels. (B) Human serum albumin (ELISA) in HBV/HDV-1cc and HBV/HDV-3cc co-infected mice at different time-points of the experiment. [file Image_3.tif]

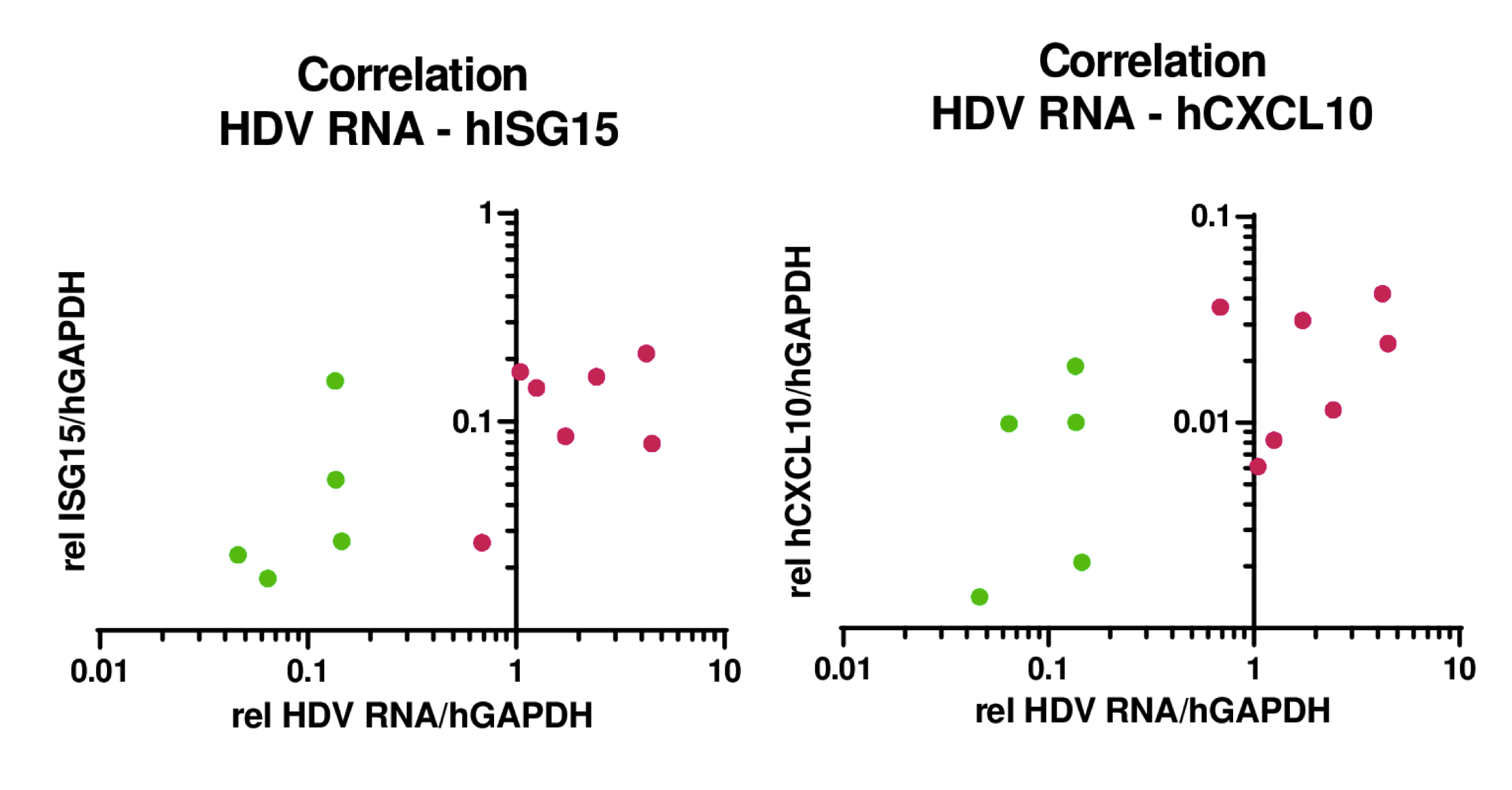

Supplement: Supplementary Figure 4 — Correlation of liver HDV RNA and hISG mRNA. qRT-PCR measurements of liver HDV RNA (normalized to housekeeping gene hGAPDH) and hISG15 or hCXCL10 (normalized to median of housekeeping genes hGAPDH and hRPL30) in HBV/HDV-3cc (red dots) and HBV/HDV-1cc infected mice (green dots) 9 weeks post infection. Correlations between HDV RNA and hISG15 or hCXCL10 show a p-value of p = 0.0323* (spearman r = 0.63) and p = 0.0591 (spearman r = 0.57), respectively, when HBV/HDV-1cc and HBV/HDV-3cc infected mice are analyzed together. [file Image_4.tiff]

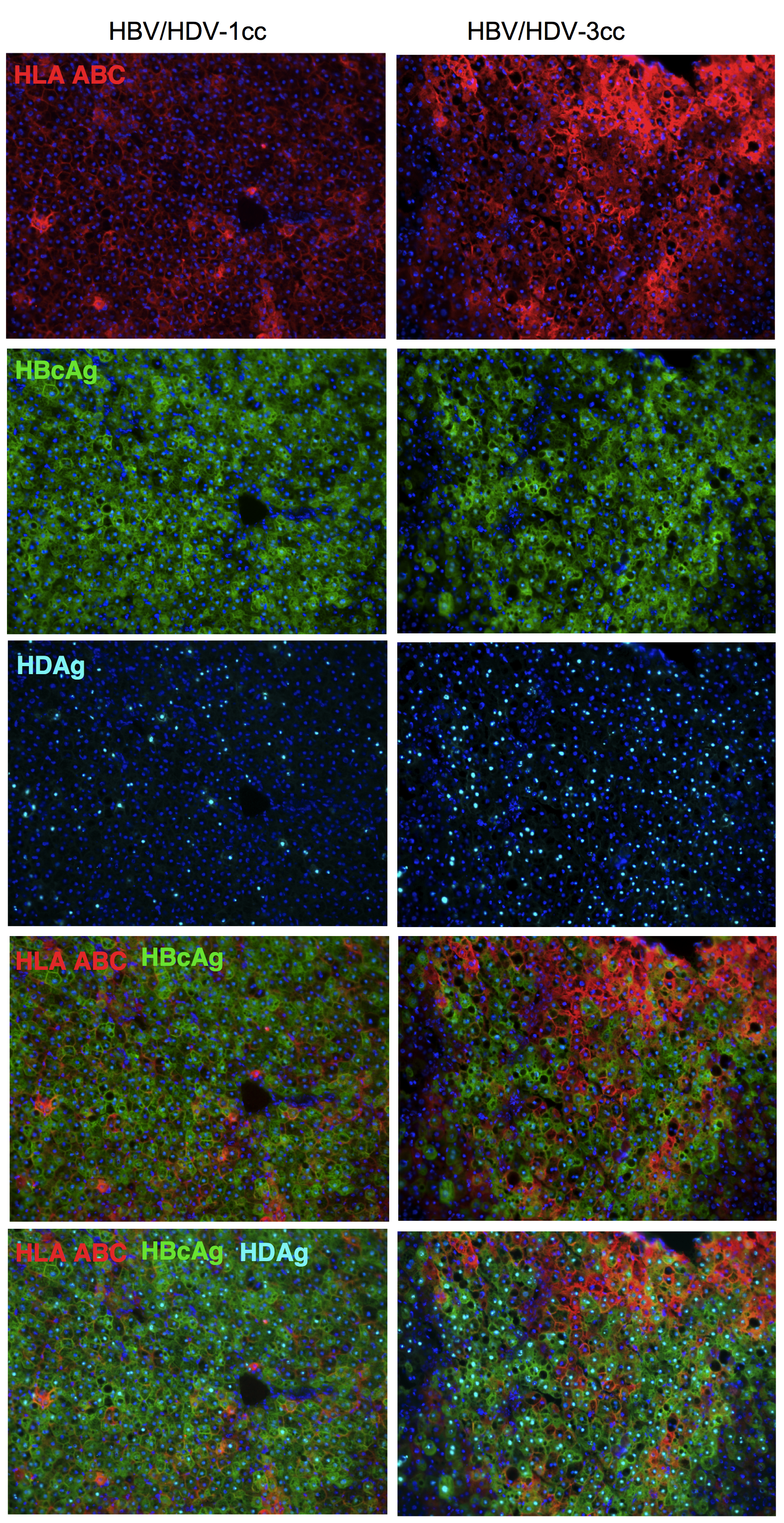

Supplement: Supplementary Figure 5 — Intrahepatic HLA ABC expression. Immunofluorescence staining of human HLA ABC (red), HBcAg (green), HDAg (turquoise) and overlays of HLA ABC with HBcAg or with HBcAg and HDAg in HBV/HDV-1cc (left) and HBV/HDV-3cc (right) co-infected mice at the end of the experiment. Nuclei are stained with Hoechst 33258 (blue). [file Image_5.tiff]

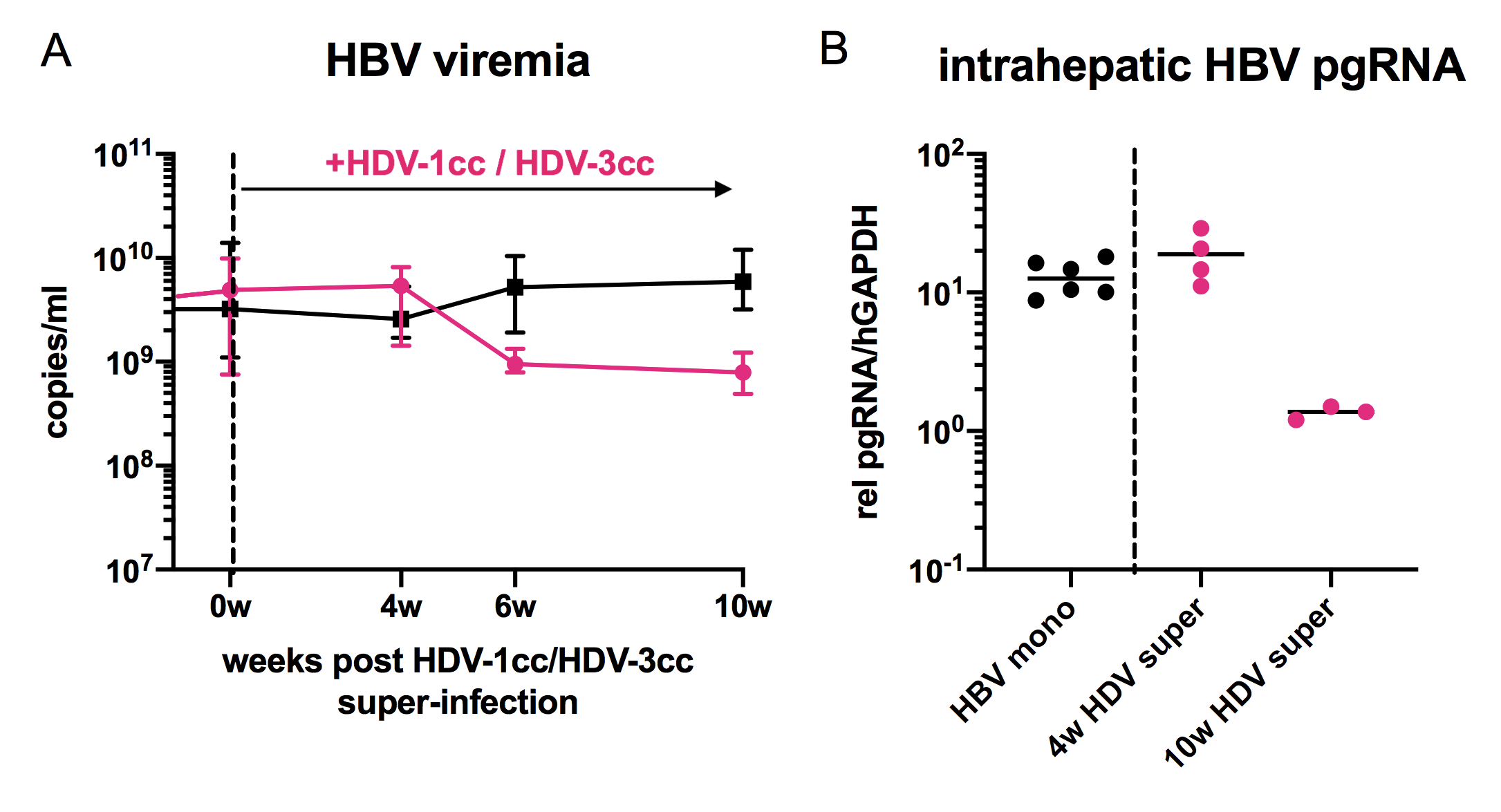

Supplement: Supplementary Figure 6 — HBV in chronic HBV-infected mice simultaneously super-infected with HDV-1cc and HDV-3cc. qRT-PCR measurements of serum HBV DNA (quantification with plasmid standard) (A) and liver pregenomic HBV RNA (normalized to housekeeping gene hGAPDH) (B) in chronic HBV-infected mice simultaneously super-infected with HDV-1cc and HDV-3cc (red line or dots) compared to stable HBV mono-infected mice (black line or dots) at indicated time-points post HDV super-infection. Results are expressed as median ± range (A), the bar shows median levels (B). [file Image_6.tiff]
